# Citrullination and PAD Enzyme Biology in Type 1 Diabetes – Regulators of Inflammation, Autoimmunity, and Pathology

**DOI:** 10.3389/fimmu.2021.678953

**Published:** 2021-06-01

**Authors:** Mei-Ling Yang, Fernanda M. C. Sodré, Mark J. Mamula, Lut Overbergh

**Affiliations:** ^1^ Section of Rheumatology, Allergy and Clinical Immunology, Department of Internal Medicine, Yale University, New Haven, CT, United States; ^2^ Department of Chronic Diseases, Metabolism and Ageing, Laboratory of Clinical and Experimental Endocrinology (CEE), KU Leuven, Leuven, Belgium

**Keywords:** type 1 diabetes, neoepitopes, post-translational modification, citrullination, peptidylarginine deiminase

## Abstract

The generation of post-translational modifications (PTMs) in human proteins is a physiological process leading to structural and immunologic variety in proteins, with potentially altered biological functions. PTMs often arise through normal responses to cellular stress, including general oxidative changes in the tissue microenvironment and intracellular stress to the endoplasmic reticulum or immune-mediated inflammatory stresses. Many studies have now illustrated the presence of ‘neoepitopes’ consisting of PTM self-proteins that induce robust autoimmune responses. These pathways of inflammatory neoepitope generation are commonly observed in many autoimmune diseases including systemic lupus erythematosus, rheumatoid arthritis, multiple sclerosis, and type 1 diabetes (T1D), among others. This review will focus on one specific PTM to self-proteins known as citrullination. Citrullination is mediated by calcium-dependent peptidylarginine deiminase (PAD) enzymes, which catalyze deimination, the conversion of arginine into the non-classical amino acid citrulline. PADs and citrullinated peptides have been associated with different autoimmune diseases, notably with a prominent role in the diagnosis and pathology of rheumatoid arthritis. More recently, an important role for PADs and citrullinated self-proteins has emerged in T1D. In this review we will provide a comprehensive overview on the pathogenic role for PADs and citrullination in inflammation and autoimmunity, with specific focus on evidence for their role in T1D. The general role of PADs in epigenetic and transcriptional processes, as well as their crucial role in histone citrullination, neutrophil biology and neutrophil extracellular trap (NET) formation will be discussed. The latter is important in view of increasing evidence for a role of neutrophils and NETosis in the pathogenesis of T1D. Further, we will discuss the underlying processes leading to citrullination, the genetic susceptibility factors for increased recognition of citrullinated epitopes by T1D HLA-susceptibility types and provide an overview of reported autoreactive responses against citrullinated epitopes, both of T cells and autoantibodies in T1D patients. Finally, we will discuss recent observations obtained in NOD mice, pointing to prevention of diabetes development through PAD inhibition, and the potential role of PAD inhibitors as novel therapeutic strategy in autoimmunity and in T1D in particular.

## Origin and Implications of Post-Translational Modifications in Autoimmunity

At a simplistic level, the success of immunity relies on distinguishing ‘self’ from ‘non-self’, originating primarily by purging the autoreactive repertoire both in central lymphoid organs, the thymus and bone marrow, as well as by peripheral tolerance mechanisms. Classical mechanisms of immune tolerance rely on the processing and presentation of self-peptides by antigen presenting cells (APCs), resulting in deletion or anergy of the autoimmune repertoire. However, it is clear that many self-proteins, or post-translational modifications (PTMs) to self-proteins, are not expressed in primary lymphoid organs. For example, PTMs specific to peripheral tissues may not be expressed in the thymus and thus these PTMs never tolerize or delete the emerging thymocytes. While we know that proteins are fundamentally assembled from 20 amino acid structures, the addition of PTMs pushes that group above 140 structurally unique amino acids (1). These observations have altered the concepts and the breadth of immune tolerance to self-proteins, particularly in the perspective of autoimmune syndromes. Many factors influence the rate at which PTMs arise in self-proteins, including the amino acid sequence, flanking amino acid motifs, and variables such as the tissue microenvironments. PTMs also alter specific peptides that arise by antigen processing and, subsequently, the specificity of ongoing B- and T-lymphocyte immunity (2–4).

Some PTMs arise by enzymatic processes, as with citrullination reviewed herein, or with N-linked glycosylation or phosphorylation, critical to biological functions of many host proteins. In contrast, some modifications arise spontaneously, under physiologic pH and temperature, such as the formation of isoaspartyl modifications [reviewed by (5)]. Also, some modifications can be processed either enzymatically or spontaneously, such as deamidation (6). As illustrated herein, PTMs may trigger aberrant autoimmunity as well as alter the biological functions of self-proteins in selected tissues, including transcriptional and translational events. The text that follows will review the conditions that elicit citrulline PTMs, including their role in biological and immunological processes, with specific focus on their implications in type 1 diabetes (T1D).

**Figure 1:**
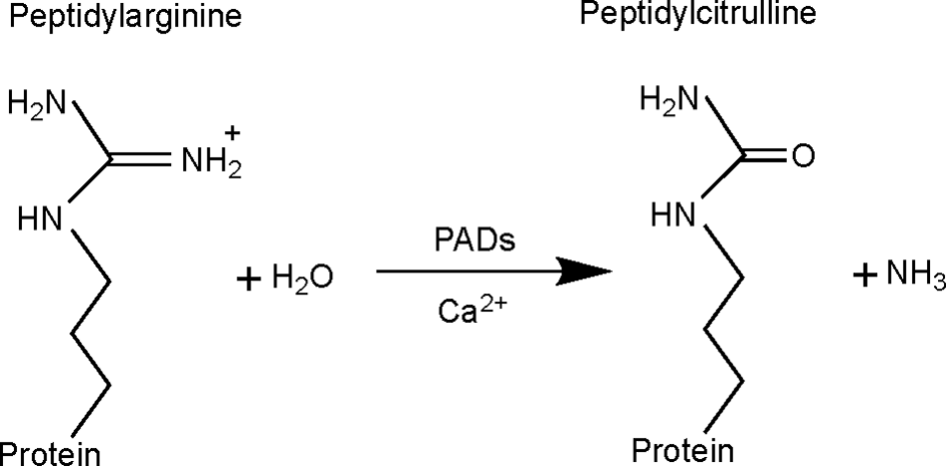
Citrullination reaction catalyzed by peptidylarginine deiminase (PAD) enzymes. With the conversion of arginine into citrulline, the primary ketamine group of arginine (=NH) is replaced by a ketone group (=O), with production of ammonia as a side-product. This results in a mass difference of 0.984 Da and loss of one positive charge. Citrullination is catalyzed by PAD enzymes, requiring Ca2^+^ as cofactor.

Citrullination, also known as deimination, is a PTM in which an arginine residue is converted into a citrulline (**Figure 1**). This modification, which was first described in 1939 (7), leads to a loss of one positive charge and a reduction in mass of 0.984 Da per modified arginine. This type of modification can alter intra- and inter-molecular interactions of the protein (8), having an impact on its structure, function and its interaction with other proteins (9). Citrullination is catalyzed by peptidylarginine deiminase (PAD) enzymes in a Ca^2+^-dependent manner (10), which is thought to be an irreversible process (11).

A role for citrullinated self-proteins has been associated with several autoimmune diseases, such as T1D, rheumatoid arthritis (RA), systemic lupus erythematosus (SLE), multiple sclerosis (MS), psoriasis, Sjögren’s syndrome (SS), antiphospholipid syndrome (ALS) and inflammatory bowel disease (IBD) (**Table 1**). Among these different autoimmune diseases, citrullination as an autoimmune biomarker in RA has been most extensively described. RA is a chronic autoimmune disease characterized by inflammation of the synovial joints. Proteomic analysis of the cellular and soluble components of RA synovium identified the full RA citrullinome, with more than 100 citrullinated proteins, amongst which vimentin, enolase, fibrinogen and fibronectin (46). Some of these were shown to induce autoantibodies and/or autoreactive T-cell responses in RA (11, 22, 29, 30). The presence of anti-citrullinated protein antibodies (ACPAs) in the serum of RA patients is one of the most specific diagnostic marker for the disease (47). ACPAs can be detected years before clinical symptoms appear (48). Apart from being a prognostic biomarker for disease development, some ACPAs have been described as useful in predicting the severity of joint destruction during the first five years after RA onset (49). ACPAs can also be detected in a small percentage of patients with SS (3 to 9.9%), and the presence of such autoantibodies indicates a risk of developing RA (50). As listed in **Table 1**, emerging citrullinated target proteins are proposed as autoimmune disease specific biomarkers. Interestingly, the serological level of citrullinated vimentin in patients with ulcerative colitis (UC) was decreased compared to patients with another important IBD, Crohn’s disease (CD), and non-IBD control subjects. Therefore, citrullinated vimentin has also been suggested as differentiating marker between CD and UC to improve the diagnostic accuracy in IBD (44).

**Table 1:** Citrullination in autoimmune diseases.

Diseases	Target proteins	References
Type 1 diabetes (T1D)	GAD65	(12–14)
	IA-2	(13)
	GRP78	(15–17)
	IGRP	(13)
	IAPP	(17, 18)
Rheumatoid arthritis (RA)	fibrinogen	(19–21)
	vimentin	(22–24)
	histone H1, H2B, H3 and H4	(25–27)
	type 2 collagen	(28)
	α-enolase	(29)
	GRP78	(30)
Systemic lupus erythematosus (SLE)	LL37	(31)
	histone H1 and H3	(26, 32)
Multiple sclerosis (MS)	MBP	(33–36)
	GFAP	(36)
	neurogranin	(37)
	histone H3	(38)
Psoriasis	keratins	(39)
	filaggrin	(40)
Sjögren’s syndrome (SS)	histone H1	(41)
	α-enolase	(42)
Antiphospholipid syndrome (ALS)	vimentin	(43)
Inflammatory bowel disease (IBD)	vimentin	(44, 45)

GAD65, glutamic acid decarboxylase 65; IA-2, islet antigen-2; GRP78, glucose-regulated protein 78; IGRP, islet-specific glucose-6-phosphatase catalytic subunit-related protein; IAPP, islet amyloid polypeptide; MBP, myelin basic protein; GFAP, glial fibrillary acidic protein.

## Peptidylarginine Deiminases: Types and Mechanism of Action

Peptidylarginine deiminases are a group of 5 enzymes encoded by genes localized on chromosome 1p36.1 in human and chromosome 4E1 in mice, located in a cluster of 350 kb and 240 kb, respectively (51). They were first described in 1977 as the enzymes mediating the conversion of arginine into the non-classical amino acid citrulline in proteins, from studies performed in mammalian hair follicles (52). The enzyme responsible for this reaction was for the first time partially purified by Fujisaki and Sugawara in 1981 and named peptidylarginine deiminase (PAD) (53). Since then, five different PAD isozymes have been described in mammals, designated as PAD1 to PAD4 and PAD6, which display 50-70% sequence identity (54). PADs replace the primary ketamine group of arginine (=NH) by a ketone group (=O) and yield ammonia as a side-product (**Figure 1**), leading to the loss of one positive charge of the target protein. This can lead to changes in the function and fate of the citrullinated target protein.

**Figure 2:**
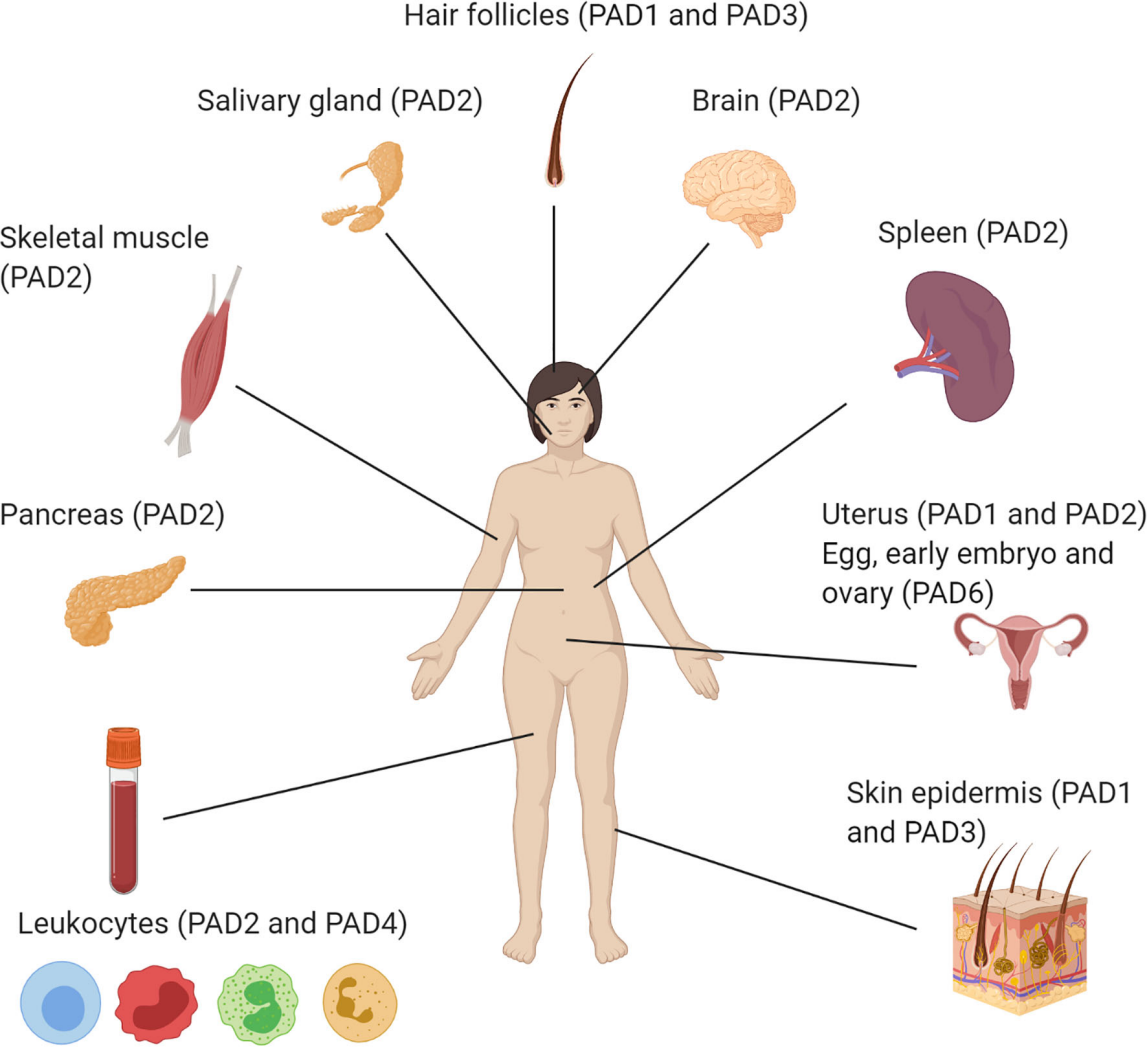
Illustration of the organ-specific protein expression of peptidylarginine deiminase isozymes in humans.

Although the PAD enzymes are widely expressed throughout the body, and have been implicated in a variety of physiological processes, each isozyme has specific tissue distribution, functions, and substrates under physiological conditions (8) (**Figure 2** and **Table 2**). As such, PAD1 is predominantly expressed in skin epidermis, uterus and hair follicles, targeting keratin and filaggrin (86). PAD2 is the ubiquitous member of the family, being expressed in multiple tissues such as brain, skeletal muscle, spleen, uterus, secretory glands and leukocytes (60, 65, 71). Importantly, PAD2 and PAD4 are the only PAD isozymes expressed in immune cells. Amongst other targets, PAD2 citrullinates myelin basic protein (MBP) in brain, vimentin in skeletal muscle and macrophages, actin in neutrophils and histones in various cell types (71). PAD3 is found in hair follicles and epidermis, and citrullinates filaggrin, trichohyalin, apoptosis-inducing factor (AIF) and vimentin (86). PAD4 is expressed in leukocytes, mainly granulocytes (like neutrophils and eosinophils), and monocytes and macrophages (63, 73). It is the only PAD isotype with a nuclear localization signal sequence, which is located at its N-terminus. PAD4 targets several nuclear proteins, such as histones, nucleophosmin and nuclear lamin C (71). With its high expression in neutrophils, PAD4 plays an important role in the generation of neutrophil extracellular traps (NETs) and thereby in the first line of defense against bacterial pathogenic invaders (see in more detail below). PAD6 is mainly expressed in eggs, embryo and ovary. It is the only PAD isozyme for which until today no protein substrates have been identified (86) and, *in vitro*, no catalytic activity can be measured (89). Also, no known association with autoimmunity or other diseases has been reported for PAD6.

**Table 2:** PAD isozymes tissue distribution, target substrates, physiological functions and disease association.

Isozyme	Tissue distribution (Protein level)	Target substrates	Physiological functions	Disease association
**PAD1**	Skin epidermis, uterus (55) and hair follicles (56)	Keratin and filaggrin (57, 58)	Skin keratinization (59)	Psoriasis (39)
**PAD2**	Brain, skeletal muscle, spleen, spinal cord, uterus, secretory glands and pancreas (55, 60–62), leukocytes [macrophages (63), neutrophils (64) and T cells (65)]	Myelin basic protein (66), vimentin (63), actin (67), histones (68), fibrinogen and α- enolase (69)	Disassembly of vimentin filaments (70), CNS plasticity (9), epigenetic and transcriptional regulation (71), immune response (65, 72)	Rheumatoid arthritis, multiple sclerosis (73), Alzheimer disease (74) and prion diseases (75)
**PAD3**	Skin epidermis and hair follicles (55, 56)	Filaggrin, trichohyalin (56, 57), apoptosis-inducing factor (76)	Regulation of epidermal functions (57)	Unknown
**PAD4**	Leukocytes [mainly granulocytes, such as neutrophils and eosinophils (77, 78), monocytes, macrophages (63) and T cells (65)] and neurons (79)	Histones, nucleophosmin (80), nuclear lamin C (81), antithrombin (82), ING4 (83), NF-Kb (84), fibrinogen and α- enolase (69)	Epigenetic and transcriptional regulation (71), NET formation (85), immune response (65, 85)	Rheumatoid arthritis, systemic lupus erythematosus, multiple sclerosis (86) and cancers (87)
**PAD6**	Egg, early embryo and ovary (88)	No substrates identified; no activity *in vitro* (89)	Oocyte cytoskeletal formation and female fertility (90)	Unknown

ING4, inhibitor of growth 4; CNS, central nervous system; NET, neutrophil extracellular trap.

Although all 5 PAD enzymes target arginine residues in proteins, they do have different substrate specificities. The basis for this difference is not fully understood, but cannot solely be explained by their difference in tissue distribution or subcellular location (54). Additional influencing factors include the enzyme kinetics, the conformation of the secondary structure of the target protein and the flanking amino acid composition surrounding the arginine residues, as revealed from *in vitro* studies with recombinant PAD enzymes. In regard to the latter, it was shown for instance that a glutamic acid accompanying the arginine residue decreases the chance of being citrullinated, whereas a flanking aspartic acid residue increases the citrullination level (91). In this regard, PAD4 was found to have a higher substrate specificity as compared to PAD2 (92, 93).

The most important regulator of PAD activity is calcium, with micro to millimolar calcium concentrations needed for their full activation (53, 54, 94). However, under physiological conditions, with intracellular cytosolic calcium concentrations usually around 100-fold lower, a basal PAD activity can also be measured, in line with the occurrence of low levels of citrullination and their role during normal physiological processes. This raises questions as to how exactly calcium is mediating the activity of PADs. One thought is that calcium may alter the confirmation of PADs. Under low calcium conditions, PADs may be present in a conformation that selects only high efficiency substrates, thus allowing citrullinations to happen, but limiting aberrant citrullination events (11). In the presence of high calcium, all calcium binding sites of the PAD enzyme are occupied, promoting extensive rearrangement and dimerization of the enzyme, leading to its full activation, as shown for PAD4 (95, 96). Alternatively or additionally, other factors are known to regulate PAD enzymes (9), which could act as cofactors modulating their calcium sensitivity and specificity (11). Considering the importance of calcium in PAD activation, pathways that alter intra- or extracellular calcium levels, such as endoplasmic reticulum (ER) and inflammatory stress, are important in many of the autoimmune diseases in which citrullination is implicated (see in more detail below) and underscore the important link between citrullination and stress pathways. Indeed, changes in calcium fluxes may lead to activation of PAD activity in stressed or dying cells. Moreover, the externalization and activation of intracellular PAD enzymes into the extracellular space may occur in the surroundings of dying cells. This may explain the citrullination of extracellular proteins, as observed for instance during NET formation (NETosis) (see in detail below) or the citrullination of beta-cell proteins as observed in T1D (15). Similar observations in RA show that PADs are highly activated by Ca^2+^ ion deposition in the inflamed joints, particularly during apoptotic cell death (97, 98), leading to elevated levels of citrullinated proteins and peptides.

## Protein Citrullination Detection

The first antibody-based methodology for citrullinated protein detection was described in 1992 by Senshu et al. (99). The antibody does not recognize citrulline residues directly but instead binds a chemically modified form, diacetyl monoxime and antipyrine derivatized-citrulline, now available in commercialized kit form. The approach utilizes protein samples separated by SDS-PAGE, transferred to a microporous membrane. The membrane bound citrulline-containing proteins are modified in the presence of 2,3-butanedione monoxime and antipyrine in acid condition. In a similar concept known as the “Senshu” method, Moelants et al. developed a sandwich ELISA format to detect citrullinated proteins utilizing antibody recognizing 2,3-butanedione-modified citrulline (100). Another citrulline-specific labeling chemical probe, rhodamine-tagged phenylglyoxal derivative (Rh-PG), was developed for detection of citrullinated proteins in both purified protein sample and complex mixtures including serum (101, 102).

There are now commercially available anti-peptidyl citrulline antibodies for detection of citrullinated proteins including mouse monoclonal IgM antibody, clone F95, from Millipore (MABN328), and rabbit polyclonal antibody from Upstate (07-377) or Abcam (ab10092 and ab6464). Clone F95 antibody is extensively used in tissue staining, immunoblot and ELISA (25, 103, 104). The specificity and sensitivity of the above commercial anti-peptidyl citrulline antibodies for differentiating arginine citrullination and lysine carbamylation were recently discussed (105). Of note, carbamylation is a non-enzymatic PTM converting lysine to homocitrulline, which shares similar structural features with citrulline. For specific citrullinated target proteins, a variety of commercially available or in-house produced antibodies are available, such as those specific to citrullinated-histones, hypoxia-inducible factor 1-α, vimentin, fibrinogen, MBP and GRP78.

Upon citrullination, 0.984 Da mass increase from the parent peptide can be identified by mass spectrometry. As this is exactly the same increase in mass as for deamidated peptides, caution is needed when analyzing liquid chromatography-tandem mass spectrometry (LC-MS/MS) data, not to misinterpret a citrullinated peptide from a deamidated peptide on a closeby N- or Q residue. On top of that, C^13^ isotopes, with an increase in mass of 1 Da compared to C^13^ parent ions, can also be wrongfully identified as deamidated or citrullinated residues, even with the use of very accurate mass spectrometry instrumentation and strict settings. A recent study by Callebaut et al. (6) outlines critical parameters to detect deamidated residues by LC-MS/MS, through minimizing artificial *in vitro* occurring deamidations and manual inspection of spectra. This same method can also be applied for critical evaluation of citrullinated peptides from LC-MS/MS analysis. Another issue to be considered is that the loss of one positive charge of the arginine residue due to citrullination will affect protease cleavage efficiency for proteases which cleave after arginine, such as trypsin. This will result in mis-cleavages by trypsin, in case the arginine is citrullinated (106). Of particular interest is the dual search delta score method developed by Qian laboratory which integrates several critical parameters for identifying citrullinated and deamidated peptides in an automated way, thereby decreasing false discovery rates (FDR) (107). A different method used for facilitating LC-MS/MS-based detection of citrullinated proteins is chemical derivatization of citrulline residues by 2,3-butanedione alone or combined with antipyrine, resulting in a mass increase of 50 or 238 Da, respectively (108, 109). Chemical derivatization of citrulline residues can also be used for enrichment of citrullinated proteins, through the use of biotin-conjugated phenylglyoxal (BPG), prior to LC-MS/MS analysis (101, 102). This method, however, requires large amounts of starting material. In addition, a BPG-based ELISA platform recently developed will validate the mass spectrometry proteomic data for citrullination detection (110).

## Inflammatory Pathways Increase Citrullination and Other PTMs

Simply put, autoimmunity is initiated when cellular and soluble components of the immune system interact to trigger the recognition and robust response to self-proteins leading to tissue pathology. Additionally, a large number of heritable genetic risk traits have been defined by genome wide association studies (GWAS) in many autoimmune syndromes. However, autoimmune syndromes and T1D, in particular, are not entirely explained by a defined collection of heritable genetic traits. Indeed, poorly defined environmental influences and epigenetic factors, which may or may not be inherited, also influence the early onset and progression of T1D (111). Of importance, the amplification of PTMs in T1D autoimmunity is clearly linked to oxidative tissue environments. Reactive oxygen species (ROS), including superoxide anion (O_2_^.-^), hydrogen radicals (OH.), and hydrogen peroxide (H_2_O_2_) are a product of a dynamic balance of endogenous anti-oxidant cellular compounds that control their tissue concentrations and biological effects. These anti-oxidants include superoxide dismutase (SOD), glutathione peroxidase, catalase, peroxiredoxins, as well as other small molecule anti-oxidants, including vitamins E and C. There are potentially a number of sources of specific ROS in tissue autoimmunity, including the infiltration of activated phagocytic cells (neutrophils, macrophages and dendritic cells) which have been demonstrated to be important in the progression and tissue pathology of T1D as well as many other autoimmune syndromes.

Oxidative stress can amplify the modification of certain proteins, or protein motifs, or, alternatively, alter metabolic pathways. As described in detail below, there are secondary effects of ROS on apoptosis, NETosis, and cellular metabolic pathways, affecting the progression of autoimmune responses and tissue pathology. ROS affected proteins may be changed in solubility, reduce the ability of proteins to be cleared, or increase immunogenicity. As detailed below, oxidation and subsequent citrullination can also provoke changes at both the level of DNA transcription and translation.

Citrullination of histone H3 by PAD4 in granulocytes, leading to formation of NETosis, is a process which is promoted by intracellular ROS (85, 112). Also, exogenous H_2_O_2_ was shown to induce citrullination and NETosis in mouse neutrophils (85). Depending on the stimulus used, the level and time course of ROS production was shown to be important for subsequent H3 citrullination and NET formation (113). However, seemingly contradictory to this notion, *in vitro* studies showed a direct inhibitory effect of PAD2 and PAD4 activity in the presence of H_2_O_2_ concentrations above 40 µM. In addition, PMA-stimulated leukocytes could effectively citrullinate recombinant fibrinogen, although this process was markedly enhanced when ROS formation was inhibited by the NADPH oxidase inhibitor diphenyleneiodonium (DPI) (114). These findings suggest that although ROS is important intracellularly for mediating citrullination of histones, supraphysiological levels of ROS may inhibit citrullination extracellularly. Discrepancies in different studies on the exact role of ROS in PAD activation and citrullination may need further examination to resolve the impact of intra- vs. extracellular ROS concentrations.

Another important factor mediating PAD activity is the local redox balance, with a reducing environment needed for efficient PAD activation. As such, *in vitro* studies, making use of the non-physiological reducing agent dithiothreitol (DTT), have shown that PADs can be activated under reducing conditions. Also more physiological reducing agents, like thioredoxin (115) and reduced glutathione (GSH) (116) can activate PAD enzymes. Kinetic characterization of PAD4 using thioredoxin as reducing agent produced results equivalent to those obtained with DTT (115).

Clearly, ER stress is a key factor in the amplification of PTMs in T1D. The insulin producing beta-cell, i.e. the immune targeted cell in T1D, is particularly sensitive to ER stress, because of its highly developed ER needed to cope with the high demands for protein translation and folding (117–119), in response to acute changes in blood glucose levels. As such, beta-cells can increase the translation of preproinsulin up to 25-fold (120), reaching almost 1 million molecules of preproinsulin per minute (121), when blood glucose levels are high. In order to restore the equilibrium between the cellular demand for protein synthesis and the ER folding capacity, cells under ER stress activate the unfolded protein response (UPR) (122). The UPR is activated by three transmembrane protein sensors, activating transcription factor 6 (ATF6), protein kinase RNA-like ER kinase (PERK) and inositol-requiring kinase 1 (IRE1). Under physiological conditions, these three sensors are kept in an inactive state through binding to the chaperone glucose-regulated protein 78 (GRP78; also known as binding immunoglobulin protein (BiP) or heat shock 70kDa protein 5 (HSPA5)) (123). When misfolded proteins accumulate in the ER lumen, GRP78 releases from these sensors inducing their activation and downstream signaling (123) leading to ER stress attenuation. When this UPR fails to restore ER homeostasis, usually in conditions of intense or chronic stress, this adaptive UPR will change to a terminal UPR (124), activating pro-apoptotic signaling pathways that lead to cell death (122).

**Figure 3:**
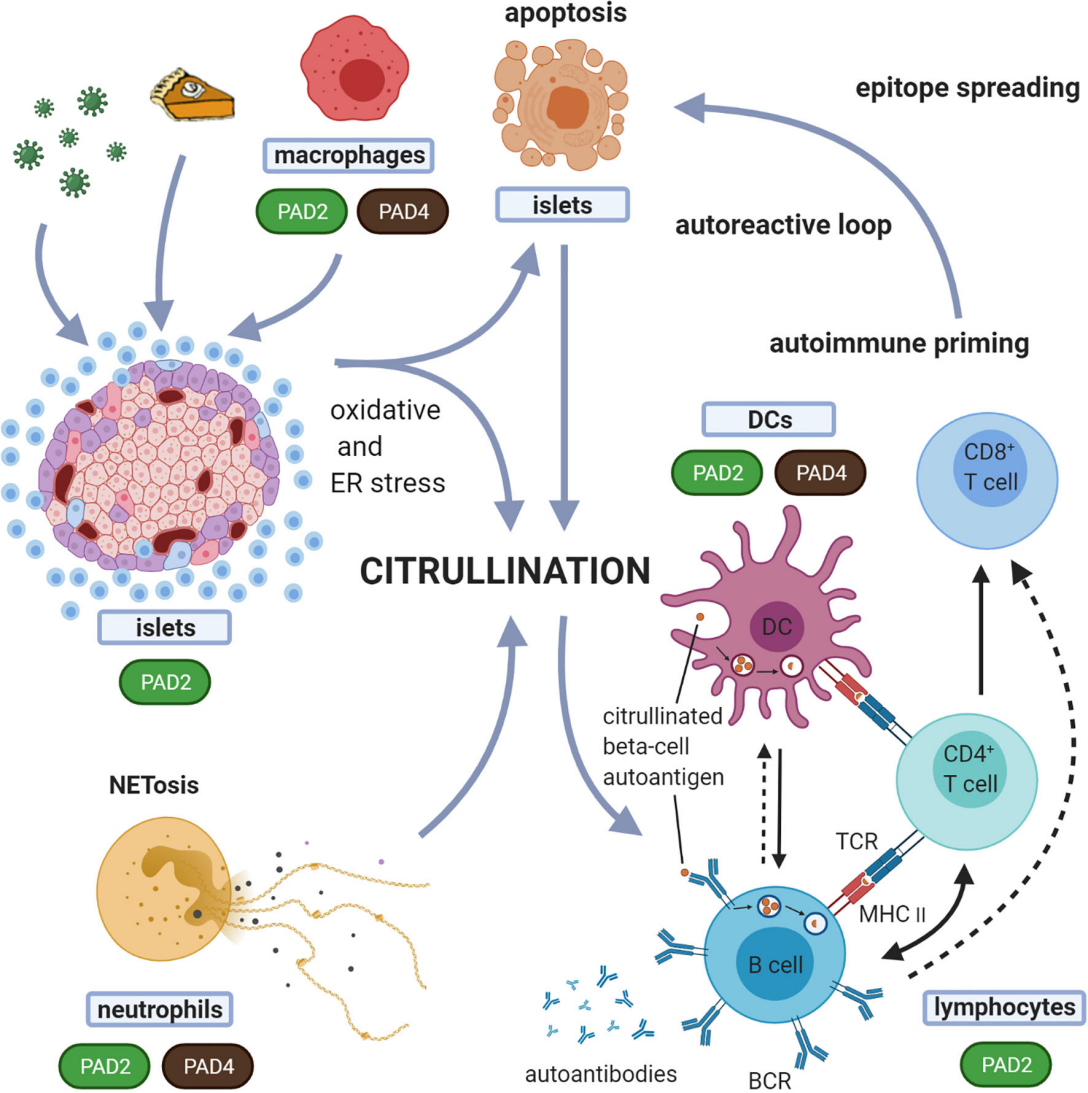
Suggested model for the role of citrullination in the induction of autoreactive responses in T1D. In this proposed model, different forms of cell death, i.e. beta-cell apoptosis and neutrophil death (NETosis), are both implicated in the generation of citrullinated proteins. Any form of environmental stress, such as viruses, inflammatory cytokines or high metabolic demand, can induce oxidative and ER stress in the beta-cells. Oxidative stress induces NETosis. Both beta-cell apoptosis and NETosis have been shown to be involved in T1D initiation and propagation. With expression and activation of PADs during stress conditions, this model suggests that both beta-cells and neutrophils, can induce citrullination. These citrullinated peptides/proteins may be processed by the immune system in different immunogenic ways, forming a source of citrullinated autoantigens. Processing of these modified proteins/peptides by antigen presenting cells, and subsequent presentation to T cells, can in turn trigger several immune responses, including activation of B cells (producing islet autoantibodies) and islet antigen-specific (effector/memory) T cells that can directly kill beta-cells presenting citrullinated islet peptides. The expression of PAD enzymes in beta-cells and neutrophils, and their activation during processes of cellular stress, underscores the relevance of this model. Dashed arrows indicate the potential interactions between B cells and CD8+ T cells and between B cells and DCs. BCR, B cell receptor; TCR, T-cell receptor.

Next to ER stress induced by high demands of protein translation, typical for secretory cells such as beta-cells, environmental factors associated with T1D can trigger additional ER stress in beta-cells (125), including coxsackie viral infection, dysglycemia, inflammation, ROS and exposure to chemicals such as streptozotocin and alloxan (125, 126). Apart from inducing beta-cell dysfunction and death, ER stress can also induce PTMs in beta-cells, a process that has been described for thapsigargin induced ER stress, showing increased activation of tissue transglutaminase (TGM2) and PAD, both Ca^2+^ dependent enzymes (127, 128). Also, inflammatory cytokines, which act at least in part through activation of ER stress pathways, were shown to induce citrullination of beta-cell proteins (15, 16, 118, 129) (**Figure 3**). Interestingly, thapsigargin induced ER stress of human beta-cells leads to increased immunogenicity, as measured by IFNγ response of T-cell clones specific for deamidated peptides (127, 128).

## Citrullinated Proteins and Their Role in Functional Pathways

### Epigenetic Role and Biological Functions of Histone Citrullination

Histones are highly basic proteins, due to the abundance of lysine and arginine residues, assembling with DNA to form nucleosomes. Changes of the positive net charge of histone due to PTMs will affect its electrostatic interaction with chromatin and chromatin accessibility such as phosphorylation, acetylation and citrullination. Of note, citrullinated histones account for about 10% of all histones in granulocytes (80). Extensive studies demonstrate that PAD catalyzed histone citrullination regulates chromatin structure (condensation versus decondensation), transcriptional regulation, and a variety of biological pathways (**Table 3**).

**Table 3:** Biological functions of histone citrullination.

Histone	PAD isozyme	Physiological or pathological roles	References
Linker H1Cit54	PAD4	impairs binding to nucleosomal DNA	(130, 131)
		chromatin decondensation in pluripotent stem cells	
H2ACit3	PAD4	occurs in calcium ionophore A23187-stimulated neutrophils	(77, 132)
H3Cit2,8,17	PAD1	facilitates early embryo genome transactivation	(133)
H3Cit2,8,17	PAD2	regulates lactation associated genes during diestrus in mammary epithelial cells	(69)
H3Cit2,8,17	PAD4	neutrophil extracellular trap (NET) formation and NETosis	(134, 135)
H3Cit8	PAD4	transcriptional repression of cytokines genes and human endogenous retroviruses (HERVs) *via* heterochromatin protein 1α (HP1α)	(136)
H3Cit26	PAD2	chromatin decondensation and transcriptional activation of estrogen receptor (ER) α-regulated gene	(68)
		potential prognostic biomarker for ER positive (ER+) breast cancer	(137)
H4Cit3	PAD4	facilitates early embryo genome transactivation	(133)
		regulates p53 pathway in apoptosis and in carcinogenesis	(81)
		neutrophil extracellular trap (NET) formation and NETosis	(12–14, 134)

H, histone; Cit, citrullination; PAD, peptidylarginine deiminase.

Histone H1 function relies on basic amino acid residues to interact with chromatin and is responsible for the formation of higher-order chromatin structure. Linker histone H1 citrullinated at residue 54 (H1Cit54; mediated by PAD4) results in activation of several pluripotency genes, such as klf2, Tcl1, Tcfap2c and Kit, due to chromatin decondensation (130, 131). Linker H1Cit54 has also been found in breast cancer cells (138) and in activated neutrophils (41). Relevant to autoimmune syndromes, autoantibodies against citrullinated linker H1 were found in about 6% of sera from patients with SLE and SS (41).

The protein core of nucleosomes is composed by core histones, H2A, H2B, H3 and H4. H2A function is finely regulated by several PTMs such as acetylation, phosphorylation and methylation (139, 140). For example, protein arginine methyltransferase 5 (PRMT5) methylates H2A at residue arginine 3 (H2AR3) which serves as an epigenetic activator to promote prostate cancer growth (140). Of note, PAD4-catalyzed citrullinated histone H2ACit3, is identified in activated neutrophils (77). Compared to other histone proteins, citrullination on histone H3 has been extensively investigated. Different PAD isozymes catalyze H3 at different arginine residues and regulate various biological functions. Both PAD1 and PAD2 citrullinate histone H3 at 2, 8 and 17 residues, H3Cit2,8,17. Citrullination of histone tails at H3R2,8,17 and H4R3 was significantly reduced in 2- and 4-cell embryos after PAD1-morpholino knockout or treatment with a PAD1 specific inhibitor. Deficiency of PAD1 resulted in mouse embryo cells arrested at the 4-cell stage (133). In human mammary epithelium cells (MCF7 cells), PAD2-catalyzed H3R2,8,17 regulates gene expression of pleotropin (PTN) and melanoma associated antigen A12 (MAGEA12) (141). In addition, PAD4-mediated H3Cit8 diminishes the binding of heterochromatin protein 1α (HP1α) to methylated histone H3K9 and leads to the suppression of gene expression of human endogenous retroviruses (HERVs) and cytokines in MCF7 cells such as TNFα, IL-1A, IL-8, IL-16 and IL-23 (136). PAD2-catalyzed H3Cit26 results in chromatin decondensation and transcriptional activation of estrogen receptor α-regulated genes in breast cancer cells (68). Therefore, H3Cit26 is believed to be a potential prognostic biomarker for estrogen receptor positive (ER+) breast cancer (137). Similar to PAD1-catalyzed H3Cit2,8,17, PAD4-catalyzed H4Cit3 also facilitates early embryo genome transactivation (133). Moreover, PAD4-catalyzed H4Cit3 regulates the p53 pathway in apoptosis and in carcinogenesis (81).

### Citrullination and Its Role in NETosis

NETosis is a cellular clearance mechanism distinct from apoptosis, which occurs when neutrophils encounter microorganisms and produce highly modified chromatin webs (142), immobilizing and killing the pathogens. The extruded DNA webs carry a number of bound bactericidal proteins (lactoferrin, elastase, proteinase 3, myeloperoxidase, cathepsin G, etc.) as well as histones and granule proteins. Thus, NETs serve as the first line defense mechanism of innate immunity to protect the host from bacteria, fungi, viruses and protozoa. Emerging evidence reveals that PTMs of histones in neutrophils regulate NETosis, and is associated with the development of autoimmune diseases such as RA, MS and SLE (**Table 3**). In leukocytes, both PAD2 and PAD4 are expressed (60), with PAD2 being mainly expressed in macrophages and PAD4 in monocytes, macrophages, eosinophils and neutrophils (63, 78). In neutrophils, citrullination is crucial for NET formation and release (85, 143). Activation of PAD4 leads to hypercitrullination of histones and consequently, decondensation and release of DNA structures coated with neutrophil granule proteins, the NETs (134, 143).

NETosis has been indicated as the major autoantigen source in SLE. NETs induce moderate levels of autoantibodies against H3Cit2, 17 and H3Cit26 in MRL/lpr mice, a spontaneous murine model of lupus (32). Moreover, autoantibodies against H1R53 were detected in patients with SLE (41). Recently, abundant citrullinated LL37 was identified in SLE target tissue (skin and kidney) and autoreactive T cells against both native and citrullinated LL37 were detected in patients with SLE, but not in RA (31). As mentioned earlier, NETosis is enhanced in RA circulating and synovial neutrophils and correlates with ACPA titers (144). H1Cit53 and H3Cit8,17,26 were found in RA and SLE neutrophil NETs (26). One study showed the presence of anti-citrullinated H2B antibodies in the anti-CCP2 positive sera from patients with RA (25). In addition, both H3Cit2,8,17 and citrullinated H4 from NETs were found as the targets of autoantibodies in patients with RA (27, 145).

The involvement of neutrophils and NETosis in T1D has been pointed out by several studies. In the non-obese diabetic (NOD) mouse, a spontaneous mouse model of autoimmune mediated beta-cell destruction and diabetes development, neutrophils and formation of NETs in the pancreas have been shown to be present during early stages of disease development and are required for diabetes development (146). In human T1D, one recent study illustrated a reduction in serum components of NETs [neutrophil elastase (NE) and proteinase 3 (PR3)], consistent with a reduced overall neutrophil count in early onset T1D (147). Conflicting studies report increases in these same NET components in T1D and a positive correlation of the circulating levels of these components with titers of autoantibodies against IA-2 and GAD65 (148). Neutrophil count is decreased in newly diagnosed T1D adult and pediatric patients (147, 149) as well as in pre-symptomatic autoantibody individuals (150). This reduction correlates with a decline in beta-cell function (151). Additionally, neutrophils infiltrate the pancreas before disease onset and during disease progression (151) and a significant fraction of these pancreas-infiltrating neutrophils forms NETs (54% and 50% in autoantibody-positive and T1D donors, respectively) (151). Relevant to citrullination, protein expression of PAD4 was elevated in neutrophils from patients with T1D and T2D (152). Given all these data indicating that neutrophils and NETosis are involved in diabetes development, the role of PADs and citrullination has also been implicated in T1D through their high expression in neutrophils and essential role in NET formation (**Figure 3**).

Of note, besides neutrophils, macrophages can release extracellular traps, a process called macrophage extracellular trap formation (METosis). The contribution of METosis in T1D has not been investigated yet, but METosis and PAD4 have been shown to contribute to self-antigen citrullination and ACPA production in autoimmune arthritis (153).

### Citrullination in Transcriptional Regulation in Immune Cells

Besides NETosis, citrullination has other functions in the immune system. In neutrophils, citrullination of NF-kB p65 enhances its nuclear translocation and transcriptional activity, increasing Toll-like receptor (TLR)-induced expression of IL-1β and TNFα (84). Citrullination of the transcription factors GATA3 and RORγt by PAD2 determines the fate of differentiating Th cells. As such, citrullination of GATA3 on R330 weakens its DNA binding ability, thereby decreasing transcription of Th2 cytokines, attenuating differentiation of Th2 cells. On the other hand, citrullination of RORγt on R56,59,77, 90 strengthens its DNA binding ability, increasing the transcription of IL-17A/F thereby enhancing the differentiation of Th17 cells (72). Also, citrullination of RNA polymerase II by PAD2 favors an efficient transcription of genes related to cellular proliferation (154). Citrullination can also reduce the potency of chemokines, such as CXCL8, CXCL10, CXCL11 and CXCL12, when compared to their native form (155–157), thereby dampening inflammatory reactions. Additionally, PADs can citrullinate the cytokine TNFα (158), reducing its capacity to stimulate the production of inflammatory chemokines, and TNFα can induce the translocation of PAD4 from the cytosol to the nucleus (38).

### Citrullinated Proteins and Their Role in Increasing Antigenicity in T1D

A primary function of the immune system is to differentiate between self and non-self-proteins. This is achieved by mechanisms that deplete the immune system of lymphocytes that react too strongly to self-antigens being present in the thymus and bone marrow, resulting in tolerance towards self-proteins. For achieving T-cell tolerance, the medullary thymic epithelial cells (mTECs) play an important role in this so-called negative selection (159). This mechanism works effectively for many self-antigens that are expressed in the thymus, through mTEC specific transcriptional regulator AIRE (autoimmune regulator), which drives expression of tissue-restricted genes such as islet specific genes, in the thymus (160). However, whether post-translationally modified self-proteins (**Tables 1** and **4**) are also expressed in the thymus has not been extensively investigated. If not, this could create a novel autoantigenic proteome for which immune tolerance has not been established in the thymus. This concept, previously described as ‘autoantigenesis’ is a term described to proteins that ‘evolve’ and acquire PTMs in a disease related target tissue, during the course of disease development, leading to B and/or autoreactive T-cell responses (4). Evidence for such antigenic modifications was shown in a mouse model of tissue-restricted self-antigen collagen type II, where the PTM reactive T cells escaped thymic selection (162). In regard to citrullination, it has been shown that PAD2 and PAD4 are expressed in murine mTECs, as measured by qPCR, thereby demonstrating that the prerequisites for negative selection of citrulline-specific T cells in the thymus are met in C57Bl6 mice (163). However, whether these enzymes are active and able to convert arginine into citrulline in proteins locally in the thymus, and whether this PAD expression and citrullination capacity is defective in autoimmune strains, such as the NOD mouse, needs further investigation.

**Table 4:** Citrullination in T1D.

Target proteins	Affected immune responses	References
GAD65	target of autoreactive T cells	(12, 13, 17)
	(HLA-A2 and HLA-DRB1*04:01)	
IAPP	target of autoreactive cells	(17, 127, 161)
	(HLA-DR and HLA-DQ8)	
IA-2	potential target of autoreactive T cells	(13)
	(HLA-A2)	
GRP78	target of autoreactive T cells	(15–17)
	(HLA-DRB1*04:01)	
	recognized by autoantibody	
IGRP	potential target of autoreactive T cells	(13)
	(HLA-A2)	

GAD65, glutamic acid decarboxylase 65; IAPP, islet amyloid polypeptide; IA-2, islet antigen-2; GRP78, glucose-regulated protein 78; IGRP, islet-specific glucose-6-phosphatase catalytic subunit-related protein.

In T1D, progressive loss of B and T-cell tolerance to beta-cell specific antigens leads to the destruction of insulin producing beta-cells. A growing number of studies suggest that immune recognition of non-conventional generated peptides/proteins (164), amongst which citrullinated proteins (12, 15, 16, 165) are an important component of that loss of tolerance (**Table 4**). As such, citrullinated proteins, generated through different stress pathways in beta-cells or neutrophils, as outlined above, could be a source of citrullinated antigens. The expression of PAD enzymes both in beta-cells and neutrophils, their activation during processes of cellular stress, and the role for both beta-cell apoptosis and NETosis in initiation and propagation of T1D, fit with such view (as schematically shown in **Figure 3**). The citrullinated peptides/proteins may be processed by the immune system in different immunogenic ways, forming a source of citrullinated autoantigens. Presentation of citrullinated proteins/peptides by antigen presenting cells, and subsequent presentation to T cells, can trigger several immune responses, including activation of B cells (producing islet autoantibodies) and islet antigen-specific (effector/memory) T cells that can directly kill beta-cells presenting citrullinated islet peptides.

Already in 1993 it was shown that the insulin B chain is prone to citrullination in the bacterial model *Porphyromonas gingivalis* (166), however, only during the last decade several publications described the link between citrullinated beta-cell proteins and autoreactive responses in T1D. In comparison with native peptides, citrullinated peptides present higher binding affinity to HLA-A2 (13) and HLA-DRB1*04:01 (12, 13, 167), diabetes-associated HLA class I and class II molecules, respectively. Of interest, there is a significant overlap in genetic susceptibility between T1D and RA, with HLA-DRB1*04:01 haplotype being a high-risk haplotype in both diseases. The antigenicity of citrullinated beta-cell antigens that bind to HLA-A2 has been demonstrated by *in vitro* activation of CD8^+^ T-cell clones, expanded from peripheral blood of HLA-A2^+^ T1D subjects, when cultured with citrullinated peptides derived from IA-2, GAD65 and IGRP (13). One citrullinated peptide of GAD65 with higher binding affinity to HLA-DRB1*04:01 was shown to be recognized by CD4^+^ T cells in the peripheral blood of T1D patients making use of *ex vivo* tetramer assays. These CD4^+^ T cells exhibited an antigen-experienced phenotype and were less- or non-responsive to the native form of the epitope (12). Shortly thereafter, Rondas et al. revealed that the ER chaperone GRP78, a major ER chaperone and a key mediator of the UPR pathway, can be citrullinated in INS-1E beta-cells under inflammatory stress, as shown by 2D-Western blotting using a citrulline specific antibody (16). This confirmed earlier proteomic studies showing an increase in PTM for GRP78 in INS-1E after IFNγ plus IL1β exposure (129). Citrullinated GRP78 was shown to be immunogenic in diabetes-prone NOD mice with appearance of both autoreactive T cells and autoantibodies (16). Furthermore, it was shown that NOD mice have significantly higher levels of *Padi2* mRNA and PAD activity in islets, already at the age of 3 weeks, as compared to normoglycemic C57Bl6 mice (16, 165). Absence of IL1β and IFNγ expression at this young age, indicated no or minor immune infiltration in the islets, suggesting expression of PAD in the endocrine cells. Further proof for this was provided in a later study, in which CD45^+^ islet infiltrating immune cells from 10-week-old NOD mice were separated from beta and alpha cells by FACS sorting. Expression of *Padi2* mRNA was evident in all 3 fractions, with comparable levels in beta-cells and CD45^+^ immune cells (Sodré et al., 2021). Further *in vitro* studies showed that GRP78 was not only citrullinated upon inflammatory stress, but was also translocated from the ER to the plasma membrane and secreted, at which level the modification of GRP78 was even much more pronounced (16, 118). This opens the view that GRP78 may become citrullinated once exposed on the plasma membrane or even after its secretion into the extracellular space. First evidence for a role of citrullinated GRP78 in human T1D, came from the discovery of a CD4^+^ T-cell clone, isolated from an islet outgrowth of a deceased T1D patient, which recognized citrullinated GRP78 epitope 292-305 (citrullinated at position 297) (17). The same study also showed autoreactive CD4^+^ T cells against citrullinated IAPP. Direct evidence for citrullination of human GRP78 at arginine residue 510 in cytokine-exposed islets was shown by targeted LC-MS/MS. Using *ex vivo* tetramer and ELISA assays, the same study showed that a subpopulation of T1D patients presents higher frequencies of CD4^+^ T cells against a citrullinated GRP78 epitope and elevated titers of autoantibodies against citrullinated GRP78 compared to healthy subjects (15). Moreover, T-cell responses and autoantibodies against citrullinated GRP78 were more frequent in long-standing T1D than in patients with new-onset T1D. Although the number of patients included in this study is too low to make hard conclusions, these last findings may indicate that immune responses against citrullinated GRP78 contribute to aggravation and/or acceleration of the disease rather than to the initiation of the disease development. Longitudinal studies on at-risk subjects are awaited to further evaluate the exact role of citrullinated antigens in disease staging.

## PTMs Can Trigger More Extensive Autoimmunity, Such as Epitope Spreading

Several investigators have identified significant differences between the T- and B-cell responses that develop against PTM self-proteins, or cryptic epitopes (5, 13, 128, 168–170). In general, T-cell responses to PTM determinants tend to be specific for the modified peptide only and more rarely cross-react with the unmodified form of the peptide. This concept was originally demonstrated in mice immunized with the isoaspartyl PTM form (isoAsp) of snRNP D lupus autoantigen protein, showing T cells only proliferate in response to the isoAsp PTM, but are unresponsive to the native (Asp) peptide (168). Alternatively, B-cell and autoantibody responses are often more promiscuous in their binding to both the PTM modified and native self-protein. This phenomenon may be due to the features of antibodies to bind flanking amino acid sequences in both modified and native protein forms. As one example, human SLE and lupus-prone MRL/lpr mice exhibit autoantibodies that bind both isoAsp and Asp forms of histone H2B (171). Moreover, it was demonstrated that autoimmune responses originating from the PTM self-protein diversify in an intra- and extra-molecular manner to other self-protein determinants. Therefore, breaking immune tolerance to a PTM self-protein promotes ‘epitope spreading’, a mechanism where autoimmunity diversifies to epitopes beyond the initial site(s) initiating the response (170). Similarly, there is both intra- and inter-molecular B- and T-cell epitope spreading in T1D, SLE, and MS (168, 172). Classical studies have described epitope spreading of autoantibody responses with the progression of autoimmune disease (173). As illustrated earlier, apoptotic and necrotic cells are rich sources of PTM-altered self-proteins in various microenvironments, notably conditions of oxidative stress (174) or altered pH (175).

In both of the collagen-induced arthritis (CIA) and experimental autoimmune encephalomyelitis (EAE) mouse models, citrullination of joint and brain proteins creates neoantigens that become additional targets in epitope spreading of autoimmune responses (172). Citrullination of aggrecan, vimentin, fibrinogen, and type II collagen, known target proteins in RA, initiates epitope spreading by promoting T-cell responses to both citrullinated peptides and the corresponding control peptides (176). In addition, citrullination-induced conformational changes of HSP90 protein unmasks cryptic epitopes to bypass B-cell tolerance in RA (177). Epitope spreading may also apply for citrullinated epitopes in triggering T1D autoimmunity (**Figure 3**). This is shown for example for citrullinated GRP78, where circulating CD4^+^ T cells and autoantibodies against both naïve and citrullinated GRP78 peptides are detectable in patients with T1D, suggesting that epitope spreading due to citrullination also occurs in T1D (15).

## PTMs in Antigen Processing and Presentation

It is clear that the specificity of ongoing immunity relies on the efficient and accurate antigen processing pathways in epitope generation (178). The presence (or absence) of a PTM of an amino acid residue has been shown to alter the recognition and cleavage by proteases. Additionally, the presence of PTM within an intracellularly processed peptide alters the binding to specific MHC motifs. For example, it has been demonstrated that the absence of N-glycosylation of the neuronal glutamate receptor subunit 3 in Rasmussen’s encephalitis, a severe form of pediatric epilepsy, exposes a granzyme B cleavage site. This is just one example of how PTMs may create a novel autoantigen (neoepitope) (179). As another example, most proteases and peptidases *do not* recognize the peptide linkage connecting isoAsp residues to its neighboring amino acid (180). Finally, Moss and coworkers demonstrated that the deamidation of asparagine residues in tetanus toxin C fragment inhibits the processing by asparagine endopeptidase and results in decreased antigen processing (181). The role of PTMs in antigen processing was examined years ago in studies of model proteins in immunity (168, 182–184). An isoaspartylated form of cytochrome c protein is cleaved differently by cathepsin D compared to the (normal) aspartyl form of the same protein (2). Granzyme B cleavage of autoantigens may also generate new epitopes based on the presence or absence of PTMs in self-protein (185). Simply put, the presence of PTMs that affect proteolytic enzyme recognition generates a completely new repertoire of peptides during antigen presentation.

Specific subsets of APCs and even the compartments within the cells in which the antigen is processed may shape the type of PTM acquired and whether modified peptide is presented on MHC. Ireland and colleagues (186) demonstrated that B-cell autophagy was required for the generation and presentation of a citrullinated peptide (but not required for the non-modified peptide form). Mamula and others have demonstrated the unique APC functions of B cells in presenting antigen to T cells. In particular, it is clear that B cells both present antigens directly, as well as transfer antigens to other APC, such as macrophages and dendritic cells. Thus, different APCs may dictate the self PTM epitopes generated and eventually presented by the immune system (187–189).

It is not fully understood as to how a PTM will affect HLA binding. The ‘fit’ of the PTM peptide versus native peptide for an HLA molecule can vary based on elements including charge and structural changes imposed by PTMs. The HLA binding of various PTM T1D autoantigens has been studied and reviewed by James et al. (12, 13). For deamidated and citrullinated residues specifically, the introduction of a negative charge enhances the binding affinity at specific positions in HLA-DR or HLA-DQ molecules. As such, HLA-DRB1*04:01 and DRB1*15:01 prefer citrulline at key positions of their binding motifs, whereas deamidation results in preferential loading onto HLA-DQ molecules (13). This was also shown for citrullinated peptides of vimentin, a RA autoantigen, which has greater affinity for HLA-DRB1*0401 than the unmodified peptide (190).

Among other autoimmune syndromes, specific PTMs of MBP result in either low, intermediate or a similar affinity for MHC compared with the corresponding wild-type peptide (191). Notably, acetylated MBP peptide (Ac 1-11) is required to incite pathogenic T cells in murine MS, though the unmodified peptide binds MHC with virtually identical kinetics. Similarly, isoaspartic acid residues in cytochrome c or snRNP D peptides (SLE autoantigens) bind MHC class II in a manner identical to the unmodified peptides (168). However, immune tolerance is maintained to the native peptides. The overall lesson is that PTM self-peptides alter processing and binding by MHC in distinct manner from unmodified (native) peptide.

## Targeting Citrullination: Types of PAD Inhibitors and Their Potential as Therapeutic Agent in Preclinical Models of Autoimmunity

**Figure 4:**
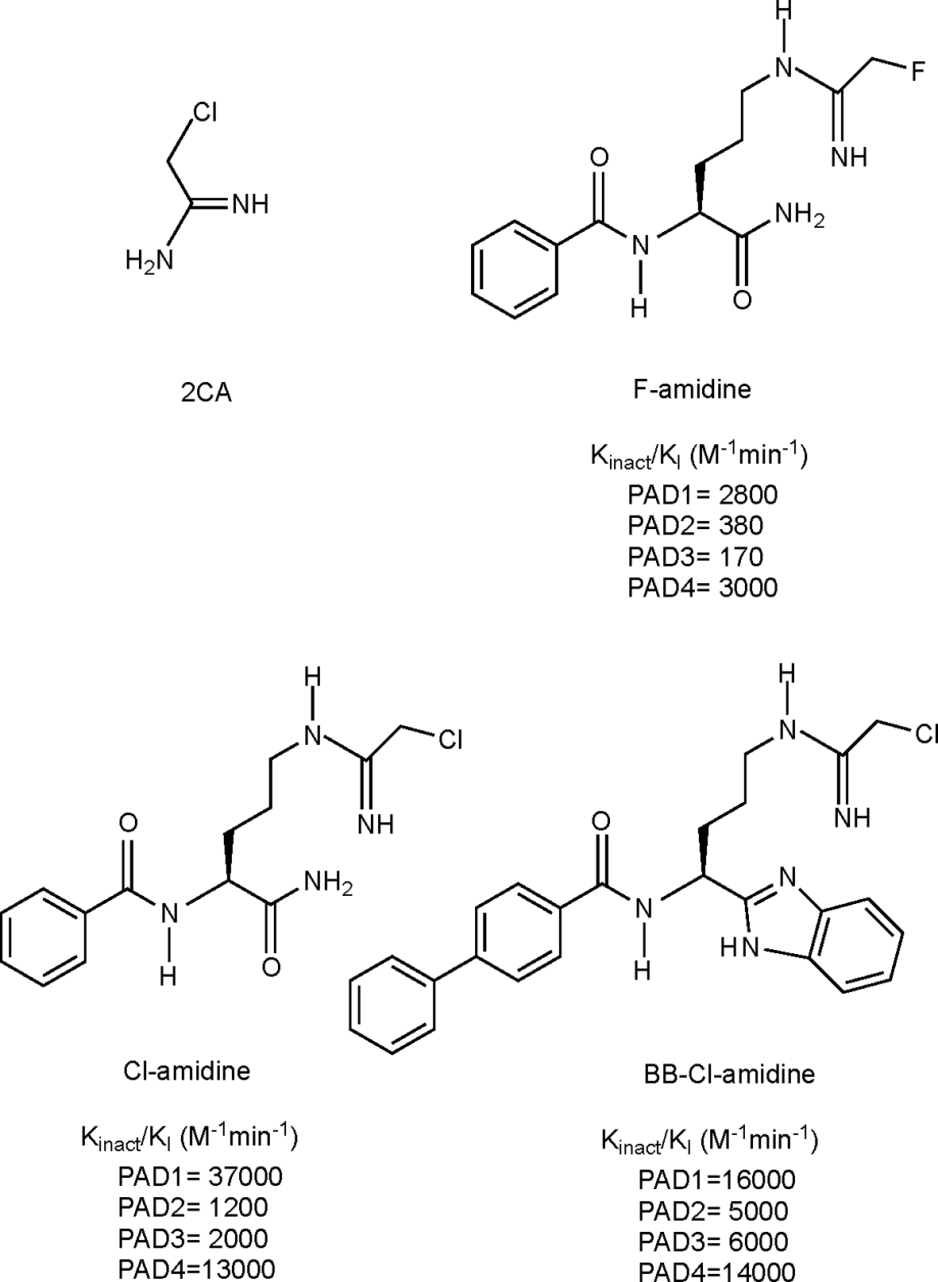
Structures and k_inact_/K_I_ values for some irreversible pan-PAD inhibitors. 2CA (193), F-amidine, Cl-amidine (194) and BB-Cl-amidine (195). k_inact_/K_I_ values have been described as the best measure of potencies for irreversible inhibitors (196). k_inact_: rate of enzyme inactivation; K_I_: inhibition constant.

Given the involvement of citrullination and PAD enzymes in several autoimmune diseases, it has been of great interest to generate compounds targeting PAD activity, i.e. PAD inhibitors. The development of such compounds was made possible with the discovery of the crystal structure of PAD enzymes (95, 192). Since then several pan-PAD inhibitors have been developed, with increasing potency, specificity and metabolic stability, of which the 4 most extensively studied irreversible inhibitors are shown in **Figure 4**. More recent efforts also include the development of specific PAD inhibitors, targeting for instance PAD2 or PAD4, as well as non-covalent reversible inhibitors. For a detailed overview of all developed PAD inhibitors we refer to a recent review specifically focusing on these developments (197).

Considering that PAD dysregulation is associated with several autoimmune diseases (86), numerous studies have evaluated the efficacy, safety and mechanism of action, mainly making use of irreversible pan-PAD inhibitors in animal models of autoimmunity, with promising results. As such, amelioration or even reversal of disease in case of intervention therapies, and delayed initiation or complete protection in case of preventive therapies, was shown in mouse models of RA, MS, SLE, UC, inflammatory bowel disease, and recently also in T1D [reviewed by (198, 199)]. In general, mechanistic insights from these studies have taught us that targeting PAD making use of pan-PAD inhibitors effectively decreases protein citrullination levels in the inflamed target tissues, as measured mainly by LC-MS/MS, PAD activity assays or Western blotting using anti-citrulline Ab (200–203). Such decreased protein citrullination can evidently have an effect on the autoreactive responses against citrullinated autoantigens, both in terms of autoantibody and T-cell responses, as shown in some published studies (200, 201). Next to this, it has become clear that other mechanisms are involved in the observed protection, pointing towards more general effects on innate and adaptive immunity. Not surprisingly, based on the high levels of PAD4 in neutrophils and their important role in histone 3 citrullination and induction of NETosis, as outlined above, *in vivo* PAD inhibition was shown to reduce NET formation and associated NET-induced tissue damage (204, 205). Other ameliorating disease effects were associated with a decrease in circulating pro-inflammatory cytokine levels, such as IL-6, TNFα and IL-1β (206, 207), an increase in Treg populations (200) or a shift in T-lymphocyte populations from Th1/Th17 towards Th2 (201, 208). The latter is thought to be mediated through direct inhibition of citrullination of the transcription factors RORγT and GATA3 (see also above) (72).

Of interest in the field of T1D, a recent study from the Overbergh laboratory showed a complete protection against diabetes development in the NOD mouse, by daily subcutaneous injections of BB-Cl-amidine (1µg/g body weight) (200). BB-Cl-amidine is a pan-PAD inhibitor that, similar to its mother compound (Cl-amidine), irreversibly inactivates PAD enzymes through covalent modification of an important cysteine for the activity of the enzymes (209). Remarkably, diabetes protection was observed when starting treatment at 8 weeks of age, a time point at which insulitis is already ongoing, but hyperglycemia has not developed (200). This observation tempts us to conclude that citrullination may play a role in amplification of the disease rather than being an initial trigger in breaking immune tolerance. BB-Cl-amidine treatment was associated with decreased citrullination levels in the pancreas as demonstrated by LC-MS/MS and western blotting with anti-citrulline Ab, as well as decreased levels of circulating autoantibodies against citrullinated GRP78, a known citrullinated autoantigen in T1D (as outlined above, **Table 4**) (15, 16). These findings confirm the effective direct action of BB-Cl-amidine on inhibition of PAD activity. Furthermore, bone-marrow derived neutrophils isolated from BB-Cl-amidine treated mice showed less potential for spontaneous NET formation when compared to the control group. Disease protection was associated with preservation of pancreatic insulin levels, although only a marginal reduction in insulitis was observed, suggesting a less aggressive form of insulitis, in line with reduced CD4^+^ effector memory T cells and reduced IFNγ-producing T cells in the pancreas infiltrates. In the periphery, a shift from Th1 towards Th2 cytokine levels and increased frequency of regulatory T cells was observed.

Taken together, the promising results obtained with pan-PAD inhibition in the NOD mouse, as well as in other autoimmune mouse models, indicate that disease protection is mediated by effective inhibition of citrullination in the target inflamed tissue, leading to decreased autoreactive responses. This further underscores the observation that citrullination is not a specific disease-related event, but rather an inflammation-dependent process occurring preferentially in autoimmune target tissues (210). Next to this, the extensive data in different preclinical autoimmune models indicate that pan-PAD inhibitors also have more general effects on immune cells, thereby dampening inflammation and reshaping the immune response towards tolerance. Whereas this may be an additional benefit for the use of PAD inhibitors, caution is also warranted. Since pan-PAD inhibitors act both intra- and extracellularly, and citrullination is important in many physiological processes, such as epigenetic and transcriptional regulation, as outlined above, the concern about possible adverse side effects cannot be neglected. Therefore, more studies are needed to evaluate more specifically potential adverse effects before translating to the human situation. Of interest in this regard is the development of isozyme-specific PAD inhibitors, which are hoped to still be protective in disease development, but allow physiological citrullination to occur. Great progress has been made in this path, with development of a high number of PAD specific inhibitors. For instance, a PAD1-selective inhibitor (211), D-Cl-amidine, and a specific PAD2 inhibitor (194), AFM30a, both based on the structure of Cl-amidine. PAD4-specific inhibitors include TDFA (212) and GSK199, with GSK199 being a reversible PAD4 inhibitor that targets the apo state of the enzyme, showing potent inhibition of PAD4 at low concentration of calcium (0.2mM) (143). GSK199 has been demonstrated to be effective in blocking murine arthritis (213), however, more studies are needed to evaluate the efficacy and safety of such specific inhibitors.

## Concluding Remarks

In summary, we have attempted to illustrate and summarize various biochemical, immunologic, and transcriptional aspects of citrulline modifications. How citrullination alters these unique biologic processes in spontaneous autoimmune syndromes are beyond the prediction of simple genetics or other stochastic factors. Citrullination, as well as many other PTMs and cellular pathways, are affected by inflammatory cytokines and reactive oxygen species that inhabit the tissue microenvironments in T1D and other autoimmune diseases. More indirect pathways are also affected by citrulline modified proteins, including downstream transcriptional events. The emerging technologies of detecting citrulline modifications as well as other PTMs in proteomics and tissue analyses will undoubtable change the landscape of autoimmunity in the coming months and years. These analyses, notably the presence of serum anti-citrulline autoantibodies, have already contributed to the clinical diagnoses and assessment of the progression of disease, and tissue pathology. With the identification of specific biomarkers and an understanding of their origins, the field will now have potential therapeutic pathways as targets to modify these autoimmune diseases, including the reducing tissue inflammation and use of inhibitors of citrullination prior to destruction of the pancreatic islets.

## Author Contributions

M-LY, FS, MM, and LO conceived the concept and co-wrote the manuscript. All authors contributed to the article and approved the submitted version.

## Funding

Research in this area in the LO lab is supported by JDRF (1-SRA-2019-809-S-B) and by IMI2-JU under grant agreement No 115797 (INNODIA) and No 945268 (INNODIA HARVEST). This Joint Undertaking receives support from the Union’s Horizon 2020 research and innovation program and “EFPIA”, ‘JDRF” and “The Leona M. and Harry B. Helmsley Charitable Trust”. M-LY and MM were supported by the JDRF (1-SRA-2020-977-S-B and 1-SRA-2020-981-S-B), NIH: AI48120-13 and DK104205-01.

## Conflict of Interest

The authors declare that the research was conducted in the absence of any commercial or financial relationships that could be construed as a potential conflict of interest.

## References

[1] Uy R Wold F. Posttranslational Covalent Modification of Proteins. Science (1977) 198:890–6. 10.1126/science.337487 337487

[2] Doyle HA Gee RJ Mamula MJ. Altered Immunogenicity of Isoaspartate Containing Proteins. Autoimmunity (2007) 40:131–7. 10.1080/08916930601165180 17453712

[3] Yang ML Gee AJP Gee RJ Zurita-Lopez CI Khare S Clarke SG. Lupus Autoimmunity Altered by Cellular Methylation Metabolism. Autoimmunity (2013) 46:21–31. 10.3109/08916934.2012.732133 23039363 PMC3543504

[4] Doyle HA Mamula MJ. Autoantigenesis: The Evolution of Protein Modifications in Autoimmune Disease. Curr Opin Immunol (2012) 24:112–8. 10.1016/j.coi.2011.12.003 PMC3288476 22209691

[5] Doyle HA Yang ML Raycroft MT Gee RJ Mamula MJ. Autoantigens: Novel Forms and Presentation to the Immune System. Autoimmunity (2014) 47:220–33. 10.3109/08916934.2013.850495 24191689

[6] Callebaut A Derua R Vig S Delong T Mathieu C Overbergh L. Identification of Deamidated Peptides in Cytokine-Exposed MIN6 Cells Through LC–MS/MS Using a Shortened Digestion Time and Inspection of MS2 Spectra. J Proteome Res (2021) 20:1405–14. 10.1021/acs.jproteome.0c00801 33372785

[7] Fearon WR. The Carbamido Diacetyl Reaction: A Test for Citrulline. Biochem J (1939) 33:902–7. 10.1042/bj0330902 PMC1264464 16746990

[8] Nicholas AP Thompson PR Bhattacharya SK. Protein Deimination in Human Health and Disease. Springer International Publishing (2017). 10.1007/978-3-319-58244-3

[9] György B Tóth E Tarcsa E Falus A Buzás EI. Citrullination: A Posttranslational Modification in Health and Disease. Int J Biochem Cell Biol (2006) 38:1662–77. 10.1016/j.biocel.2006.03.008 16730216

[10] Takahara H Okamoto H Sugawara K. Calcium-Dependent Properties of Peptidylarginine Deiminase From Rabbit Skeletal Muscle. Agric Biol Chem (1986) 50:2899–904. 10.1080/00021369.1986.10867830

[11] Darrah E Andrade F. Rheumatoid Arthritis and Citrullination. Curr Opin Rheumatol (2018) 176:139–48. 10.1097/BOR.0000000000000452 PMC5848217 28937414

[12] McGinty JW Chow IT Greenbaum C Odegard J Kwok WW James EA. Recognition of Posttranslationally Modified GAD65 Epitopes in Subjects With Type 1 Diabetes. Diabetes (2014) 63:3033–40. 10.2337/db13-1952 PMC4392921 24705406

[13] McGinty JW Marré ML Bajzik V Piganelli JD James EA. T Cell Epitopes and Post-Translationally Modified Epitopes in Type 1 Diabetes. Curr Diabetes Rep (2015) 15:1–14. 10.1007/s11892-015-0657-7 PMC4902156 26370701

[14] Reijonen H Novak EJ Kochik S Heninger A Liu AW Kwok WW. Detection of GAD65-specific T-Cells by Major Histocompatibility Complex Class II Tetramers in Type 1 Diabetic Patients and At-Risk Subjects. Diabetes (2002) 51:1375–82. 10.2337/diabetes.51.5.1375 11978633

[15] Buitinga M Callebaut A Marques Câmara Sodré F Crèvecoeur I Blahnik-Fagan G Yang M. Inflammation-Induced Citrullinated Glucose-Regulated Protein 78 Elicits Immune Responses in Human Type 1 Diabetes. Diabetes (2018) 67:2337–48. 10.2337/db18-0295 PMC6973547 30348823

[16] Rondas D Crèvecoeur I D’Hertog W Ferreira GB Staes A Garg AD. Citrullinated Glucose-Regulated Protein 78 Is an Autoantigen in Type 1 Diabetes. Diabetes (2015) 64:573–86. 10.2337/db14-0621 25204978

[17] Babon JAB Denicola ME Blodgett DM Crèvecoeur I Buttrick TS Maehr R. Analysis of Self-Antigen Specificity of Islet-Infiltrating T Cells From Human Donors With Type 1 Diabetes. Nat Med (2016) 22:1482–7. 10.1038/nm.4203 PMC5140746 27798614

[18] Denroche HC Verchere CB. IAPP and Type 1 Diabetes: Implications for Immunity, Metabolism and Islet Transplants. J Mol Endocrinol (2018) 60:R57–75. 10.1530/JME-17-0138 29378867

[19] Takizawa Y Suzuki A Sawada T Ohsaka M Inoue T Yamada R. Citrullinated Fibrinogen Detected as a Soluble Citrullinated Autoantigen in Rheumatoid Arthritis Synovial Fluids. Ann Rheum Dis (2006) 65:1013–20. 10.1136/ard.2005.044743 PMC1798256 16449316

[20] Raijmakers R van Beers JJBC El-Azzouny M Visser NFC Božič B Pruijn GJM. Elevated Levels of Fibrinogen-Derived Endogenous Citrullinated Peptides in Synovial Fluid of Rheumatoid Arthritis Patients. Arthritis Res Ther (2012) 14:1–10. 10.1186/ar3840 22584083 PMC3446491

[21] Zhao X Okeke NL Sharpe O Batliwalla FM Lee AT Ho PP. Circulating Immune Complexes Contain Citrullinated Fibrinogen in Rheumatoid Arthritis. Arthritis Res Ther (2008) 10:1–13. 10.1186/ar2478 PMC2575608 18710572

[22] Feitsma AL Van Der Voort EIH Franken KLMC El Bannoudi HE Elferink BG Drijfhout JW. Identification of Citrullinated Vimentin Peptides as T Cell Epitopes in HLA-DR4-Positive Patients With Rheumatoid Arthritis. Arthritis Rheum (2010) 62:117–25. 10.1002/art.25059 20039411

[23] Tilleman K Van Steendam K Cantaert T De Keyser F Elewaut D Deforce D. Synovial Detection and Autoantibody Reactivity of Processed Citrullinated Isoforms of Vimentin in Inflammatory Arthritides. Rheumatology (2008) 47:597–604. 10.1093/rheumatology/ken077 18326534

[24] Steendam KV Tilleman K Ceuleneer M Keyser F Elewaut D Deforce D. Citrullinated Vimentin as an Important Antigen in Immune Complexes From Synovial Fluid of Rheumatoid Arthritis Patients With Antibodies Against Citrullinated Proteins. Arthritis Res Ther (2010) 12:R132. 10.1186/ar3070 20609218 PMC2945022

[25] Sohn DH Rhodes C Onuma K Zhao X Sharpe O Gazitt T. Local Joint Inflammation and Histone Citrullination in a Murine Model of the Transition From Preclinical Autoimmunity to Inflammatory Arthritis. Arthritis Rheumatol (2015) 67:2877–87. 10.1002/art.39283 PMC4626401 26227989

[26] Chapman EA Lyon M Simpson D Mason D Beynon RJ Moots RJ. Caught in a Trap? Proteomic Analysis of Neutrophil Extracellular Traps in Rheumatoid Arthritis and Systemic Lupus Erythematosus. Front Immunol (2019) 10:423. 10.3389/fimmu.2019.00423 30915077 PMC6421309

[27] Janssen KMJ de Smit MJ Withaar C Brouwer E van Winkelhoff AJ Vissink A. Autoantibodies Against Citrullinated Histone H3 in Rheumatoid Arthritis and Periodontitis Patients. J Clin Periodontol (2017) 44:577–84. 10.1111/jcpe.12727 28370244

[28] Yoshida M Tsuji M Kurosaka D Kurosaka D Yasuda J Ito Y. Autoimmunity to Citrullinated Type II Collagen in Rheumatoid Arthritis. Mod Rheumatol (2006) 16:276–81. 10.1007/s10165-006-0498-y PMC2780673 17039307

[29] Gerstner C Dubnovitsky A Sandin C Kozhukh G Uchtenhagen H James EA. Functional and Structural Characterization of a Novel HLA-DRB1*04: 01-Restricted α-Enolase T Cell Epitope in Rheumatoid Arthritis. Front Immunol (2016) 7:494. 10.3389/fimmu.2016.00494 27895642 PMC5108039

[30] Shoda H Fujio K Shibuya M Okamura T Sumitomo S Okamoto A. Detection of Autoantibodies to Citrullinated BiP in Rheumatoid Arthritis Patients and Pro-Inflammatory Role of Citrullinated BiP in Collagen-Induced Arthritis. Arthritis Res Ther (2011) 13(R191):1–12. 10.1186/ar3520 PMC3334641 22108001

[31] Lande R Palazzo R Gestermann N Jandus C Falchi M Spadaro F. Native/Citrullinated LL37-Specific T-Cells Help Autoantibody Production in Systemic Lupus Erythematosus. Sci Rep (2020) 10:1–14. 10.1038/s41598-020-62480-3 32245990 PMC7125190

[32] Liu CL Tangsombatvisit S Rosenberg JM Mandelbaum G Gillespie EC Gozani OP. Specific Post-Translational Histone Modifications of Neutrophil Extracellular Traps as Immunogens and Potential Targets of Lupus Autoantibodies. Arthritis Res Ther (2012) 14:1–14. 10.1186/ar3707 22300536 PMC3392818

[33] Moscarello MA Wood DD Ackerley C Boulias C. Myelin in Multiple Sclerosis Is Developmentally Immature. J Clin Invest (1994) 94:164–54. 10.1172/JCI117300 PMC296292 7518827

[34] Wood DD Bilbao JM Connors PO Moscarello MA. Acute Mul Tiple Sclerosis (Marburg Type) Is Associated With Developmentally Immature Myelin Basic Protein. Ann Neurol (1996) 40:18–24. 10.1002/ana.410400106 8687186

[35] Deraos G Chatzantoni K Matsoukas M Tselios T Deraos S Katsara M. Citrullination of Linear and Cyclic Altered Peptide Ligands From Myelin Basic Protein (Mbp 87 - 99) Epitope Elicits a Th1 Polarized Response by T Cells Isolated From Multiple Sclerosis Patients: Implications in Triggering Disease. J Med Chem (2008) 51:7834–42. 10.1021/jm800891n 19053745

[36] Bradford CM Ramos I Cross AK Haddock G McQuaid S Nicholas AP. Localisation of Citrullinated Proteins in Normal Appearing White Matter and Lesions in the Central Nervous System in Multiple Sclerosis. J Neuroimmunol (2014) 273:85–95. 10.1016/j.jneuroim.2014.05.007 24907905

[37] Faigle W Cruciani C Wolski W Roschitzki B Puthenparampil M Tomas-Ojer P. Brain Citrullination Patterns and T Cell Reactivity of Cerebrospinal Fluid-Derived CD4+ T Cells in Multiple Sclerosis. Front Immunol (2019) 10:540. 10.3389/fimmu.2019.00540 31024521 PMC6467957

[38] Mastronardi FG Wood DD Mei J Raijmakers R Tseveleki V Dosch HM. Increased Citrullination of Histone H3 in Multiple Sclerosis Brain and Animal Models of Demyelination: A Role for Tumor Necrosis Factor-Induced Peptidylarginine Deiminase 4 Translocation. J Neurosci (2006) 26:11387–96. 10.1523/JNEUROSCI.3349-06.2006 PMC6674531 17079667

[39] Ishida-Yamamoto A Senshu T Takahashi H Akiyama K Nomura K Iizuka H. Decreased Deiminated Keratin K1 in Psoriatic Hyperproliferative Epidermis. J Invest Dermatol (2000) 114:701–5. 10.1046/j.1523-1747.2000.00936.x 10733676

[40] Senshu T Akiyama K Kan S Asaga H Ishigami A Manabe M. Detection of Deiminated Proteins in Rat Skin: Probing With a Monospecific Antibody After Modification of Citrulline Residues. J Invest Dermatol (1995) 105:163–9. 10.1111/1523-1747.ep12317070 7543546

[41] Dwivedi N Neeli I Schall N Wan H Desiderio DM Csernok E. Deimination of Linker Histones Links Neutrophil Extracellular Trap Release With Autoantibodies in Systemic Autoimmunity. FASEB J (2014) 28:2840–51. 10.1096/fj.13-247254 PMC4756806 24671707

[42] Nezos A Cinoku I Mavragani CP Moutsopoulos HM. Antibodies Against Citrullinated Alpha Enolase Peptides in Primary Sjogren’s Syndrome. Clin Immunol (2017) 183:300–3. 10.1016/j.clim.2017.09.012 28919520

[43] Alessandri C Agmon-Levin N Conti F Perricone C Ortona E Pendolino M. Anti-Mutated Citrullinated Vimentin Antibodies in Antiphospholipid Syndrome: Diagnostic Value and Relationship With Clinical Features. Immunol Res (2017) 65:524–31. 10.1007/s12026-017-8899-x 28215033

[44] Mortensen JH Godskesen E Dam M Haaften TV Klinge G Olinga P. Fragments of Citrullinated and MMP- Degraded Vimentin and MMP-degraded Type III Collagen Are Novel Serological Biomarkers to Differentiate Crohn‘s Disease From Ulcerative Colitis. J Crohn’s Colitis (2015) 9:863–72. 10.1093/ecco-jcc/jjv123 26188349

[45] Al-Jarallah K Shehab D Al-Attiyah R Al-Azmi W Al-Fadli A Zafar Haider M. Antibodies to Mutated Citrullinated Vimentin and Anti-Cyclic Citrullinated Peptide Antibodies in Inflammatory Bowel Disease and Related Arthritis. Inflammation Bowel Dis (2012) 18:1655–62. 10.1002/ibd.21937 22114016

[46] Wang F Chen F Gao W Wang H Zhao N Xu M. Identification of Citrullinated Peptides in the Synovial Fluid of Patients With Rheumatoid Arthritis Using LC-MALDI-TOF/TOF. Clin Rheumatol (2016) 35:2185–94. 10.1007/s10067-016-3247-4 PMC4989008 27060082

[47] Aletaha D Neogi T Silman AJ Funovits J Felson DT Bingham CO. 2010 Rheumatoid Arthritis Classification Criteria: An American College of Rheumatology/European League Against Rheumatism Collaborative Initiative. Arthritis Rheum (2010) 62:2569–81. 10.1002/art.27584 20872595

[48] Rantapää-Dahlqvist S Jong BAW De Berglin E Stenlund H Sundin U. Venrooij WJ Van. Antibodies Against Cyclic Citrullinated Peptide and IgA Rheumatoid Factor Predict the Development of Rheumatoid Arthritis. Arthritis Rheum (2003) 48:2741–9. 10.1002/art.11223 14558078

[49] Meyer O Labarre C Dougados M Goupille P Cantagrel A Dubois A. Anticitrullinated Protein/Peptide Antibody Assays in Early Rheumatoid Arthritis for Predicting Five Year Radiographic Damage. Ann Rheum Dis (2003) 62:120–6. 10.1136/ard.62.2.120 PMC1754441 12525380

[50] Molano-González N Olivares-Martínez E Anaya JM Hernández-Molina G. Anti-Citrullinated Protein Antibodies and Arthritis in Sjögren’s Syndrome: A Systematic Review and Meta-Analysis. Scand J Rheumatol (2019) 48:157–63. 10.1080/03009742.2018.1469164 30270696

[51] Chavanas S Méchin MC Takahara H Kawada A Nachat R Serre G. Comparative Analysis of the Mouse and Human Peptidylarginine Deiminase Gene Clusters Reveals Highly Conserved Non-Coding Segments and a New Human Gene, PADI6. Gene (2004) 330:19–27. 10.1016/j.gene.2003.12.038 15087120

[52] Rogers GE Harding HWJ Llewellyn-Smith IJ. The Origin of Citrulline-Containing Proteins in the Hair Follicle and the Chemical Nature of Trichohyalin, an Intracellular Precursor. BBA - Protein Struct (1977) 495:159–75. 10.1016/0005-2795(77)90250-1 410454

[53] Fujisaki M Sugawara K. Properties Epidermis of Peptidylarginine of Newborn Rats. J Biochem (1981) 89:257–63. 10.1093/oxfordjournals.jbchem.a133189 7217033

[54] Amin B Voelter W. Human Deiminases: Isoforms, Substrate Specificities, Kinetics, and Detection. Prog Chem Org Nat Prod (2017) 106:203–40. 10.1007/978-3-319-93506-5 28762090

[55] Terakawa H Takahara H Sugawara K. Three Types of Mouse Peptidylarginine Deiminase: Characterization and Tissue Distribution. J Biochem (1991) 110:661–6. 10.1093/oxfordjournals.jbchem.a123636 1778991

[56] Nachat R Méchin MC Charveron M Serre G Constans J Simon M. Peptidylarginine Deiminase Isoforms Are Differentially Expressed in the Anagen Hair Follicles and Other Human Skin Appendages. J Invest Dermatol (2005) 125:34–41. 10.1111/j.0022-202X.2005.23763.x 15982300

[57] Nachat R Méchin MC Takahara H Chavanas S Charveron M Serre G. Peptidylarginine Deiminase Isoforms 1-3 Are Expressed in the Epidermis and Involved in the Deimination of K1 and Filaggrin. J Invest Dermatol (2005) 124:384–93. 10.1111/j.0022-202X.2004.23568.x 15675958

[58] Senshu T Kan S Ogawa H Manabe M Asaga H. Preferential Deimination of Keratin K1 and Filaggrin During the Terminal Differentiation of Human Epidermis. Biochem Biophys Res Commun (1996) 719:712–9. 10.1006/bbrc.1996.1240 8780679

[59] Ishida-Yamamoto A Senshu T Eady RAJ Takahashi H Shimizu H Akiyama M. Sequential Reorganization of Cornified Cell Keratin Filaments Involving Filaggrin-Mediated Compaction and Keratin 1 Deimination. J Invest Dermatol (2002) 118:282–7. 10.1046/j.0022-202x.2001.01671.x 11841545

[60] Beers JJBCV Zendman AJW Raijmakers R Stammen-vogelzangs J Pruijn GJM. Peptidylarginine Deiminase Expression and Activity in PAD2 Knock-Out and PAD4-Low Mice. Biochimie (2013) 95:299–308. 10.1016/j.biochi.2012.09.029 23026755

[61] Takahara H Tsuchida M Kusubata M Akutsu K Tagami Sugawara SK. Peptidylarginine Deiminase of the Mouse. Distribution, Properties, and Immunocytochemical Localization. J Biol Chem (1989) 264:13361–8. 10.1016/S0021-9258(18)51637-9 2753915

[62] Watanabe K Akiyama K Hikichi K Ohtsuka R Okuyama A Senshu T. Combined Biochemical and Immunochemical Comparison of Peptidylarginine Deiminases Present in Various Tissues. BBA - Gen Subj (1988) 966:375–83. 10.1016/0304-4165(88)90088-8 3416014

[63] Vossenaar ER Radstake TRD Van Der Heijden A Van Mansum MAM Dieteren C De Rooij DJ. Expression and Activity of Citrullinating Peptidylarginine Deiminase Enzymes in Monocytes and Macrophages. Ann Rheum Dis (2004) 63:373–81. 10.1136/ard.2003.012211 PMC1754951 15020330

[64] Zhou Y Chen B Mittereder N Chaerkady R Strain M An LL. Spontaneous Secretion of the Citrullination Enzyme PAD2 and Cell Surface Exposure of PAD4 by Neutrophils. Front Immunol (2017) 8:1200. 10.3389/fimmu.2017.01200 28993780 PMC5622307

[65] Liu Y Lightfoot YL Seto N Carmona-Rivera C Moore E Goel R. Peptidylarginine Deiminases 2 and 4 Modulate Innate and Adaptive Immune Responses in TLR-7-Dependent Lupus. JCI Insight (2018) 3:1–21. 10.1172/jci.insight.124729 PMC6328098 30518690

[66] Lamensa JWE Moscarello MA. Deimination of Human Myelin Basic Protein by a Peptidylarginine Deiminase From Bovine Brain. J Neurochem (1993) 61:987–96. 10.1111/j.1471-4159.1993.tb03612.x 7689646

[67] Darrah E Rosen A Giles JT Andrade F. Peptidylarginine Deiminase 2, 3 and 4 Have Distinct Specificities Against Cellular Substrates: Novel Insights Into Autoantigen Selection in Rheumatoid Arthritis. Ann Rheum Dis (2012) 71:92–8. 10.1136/ard.2011.151712 PMC3302156 21859690

[68] Zhang X Bolt M Guertin MJ Chen W Zhang S Cherrington BD. Peptidylarginine Deiminase 2-Catalyzed Histone H3 Arginine 26 Citrullination Facilitates Estrogen Receptor α Target Gene Activation. Proc Natl Acad Sci (2012) 109:13331–6. 10.1073/pnas PMC3421185 22853951

[69] Damgaard D Bawadekar M Senolt L Stensballe A Shelef MA Nielsen CH. Relative Efficiencies of Peptidylarginine Deiminase 2 and 4 in Generating Target Sites for Anti-Citrullinated Protein Antibodies in Fibrinogen, Alpha-Enolase and Histone H3. PloS One (2018) 13:1–14. 10.1371/journal.pone.0203214 PMC6117052 30161253

[70] Inagaki M Takahara H Nishi Y Sugawara K Sato C. Ca2+-Dependent Deimination-Induced Dissembly of Intermediate Filaments Involves Specific Modification of the Amino-Terminal Head Domain. J Biol Chem (1989) 264:18119–27. 10.1016/S0021-9258(19)84685-9 2808368

[71] Wang S Wang Y. Peptidylarginine Deiminases in Citrullination, Gene Regulation, Health and Pathogenesis. Biochim Biophys Acta - Gene Regul Mech (2013) 1829:1126–35. 10.1016/j.bbagrm.2013.07.003 PMC3775966 23860259

[72] Sun B Chang HH Salinger A Tomita B Bawadekar M Holmes CL. Reciprocal Regulation of Th2 and Th17 Cells by PAD2-Mediated Citrullination. JCI Insight (2019) 4:e129687. 10.1172/jci.insight.129687 PMC6948856 31723060

[73] Vossenaar ER Zendman AJW Van Venrooij WJ Pruijn GJM. PAD, a Growing Family of Citrullinating Enzymes: Genes, Features and Involvement in Disease. BioEssays (2003) 25:1106–18. 10.1002/bies.10357 14579251

[74] Ishigami A Ohsawa T Hiratsuka M Taguchi H Kobayashi S Saito Y. Abnormal Accumulation of Citrullinated Proteins Catalyzed by Peptidylarginine Deiminase in Hippocampal Extracts From Patients With Alzheimer’s Disease. J Neurosci Res (2005) 80:120–8. 10.1002/jnr.20431 15704193

[75] Jang B Jin JK Jeon YC Cho HJ Ishigami A Choi KC. Involvement of Peptidylarginine Deiminase-Mediated Post-Translational Citrullination in Pathogenesis of Sporadic Creutzfeldt-Jakob Disease. Acta Neuropathol (2010) 119:199–210. 10.1007/s00401-009-0625-x 20013286

[76] KP U Subramanian V Nicholas AP Thompson PR Ferretti P. Modulation of Calcium-Induced Cell Death in Human Neural Stem Cells by the Novel Peptidylarginine Deiminase-AIF Pathway. Biochim Biophys Acta - Mol Cell Res (2014) 1843:1162–71. 10.1016/j.bbamcr.2014.02.018 PMC3996523 24607566

[77] Nakashima K Hagiwara T Yamada M. Nuclear Localization of Peptidylarginine Deiminase V and Histone Deimination in Granulocytes. J Biol Chem (2002) 277:49562–8. 10.1074/jbc.M208795200 12393868

[78] Asaga H Nakashima K Senshu T Ishigami A Yamada M. Immunocytochemical Localization of Peptidylarginine Deiminase in Human Eosinophils and Neutrophils. J Leukoc Biol (2001) 70:46–51. 10.1189/jlb.70.1.46 11435484

[79] Acharya NK Nagele EP Han M Coretti NJ Demarshall C Kosciuk MC. Neuronal PAD4 Expression and Protein Citrullination : Possible Role in Production of Autoantibodies Associated With Neurodegenerative Disease. J Autoimmun (2012) 38:369–80. 10.1016/j.jaut.2012.03.004 22560840

[80] Hagiwara T Nakashima K Hirano H Senshu T Yamada M. Deimination of Arginine Residues in Nucleophosmin/B23 and Histones in HL-60 Granulocytes. Biochem Biophys Res Commun (2002) 290:979–83. 10.1006/bbrc.2001.6303 11798170

[81] Tanikawa C Espinosa M Suzuki A Masuda K Yamamoto K Tsuchiya E. Regulation of Histone Modification and Chromatin Structure by the P53-PADI4 Pathway. Nat Commun (2012) 3:676. 10.1038/ncomms1676 22334079

[82] Chang X Yamada R Sawada T Suzuki A Kochi Y Yamamoto K. The Inhibition of Antithrombin by Peptidylarginine Deiminase 4 may Contribute to Pathogenesis of Rheumatoid Arthritis. Rheumatology (2005) 44:293–8. 10.1093/rheumatology/keh473 15561738

[83] Guo Q Fast W. Citrullination of Inhibitor of Growth 4 (ING4) by Peptidylarginine Deminase 4 (PAD4) Disrupts the Interaction Between ING4 and p53*. J Biol Chem (2011) 286:17069–78. 10.1074/jbc.M111.230961 PMC3089551 21454715

[84] Sun B Dwivedi N Bechtel TJ Paulsen JL Muth A Bawadekar M. Citrullination of NF-κb p65 Promotes Its Nuclear Localization and TLR-Induced Expression of IL-1β and Tnfα. Sci Immunol (2017) 2:eaal3062. 10.1016/j.physbeh.2017.03.040 28783661 PMC5718838

[85] Li P Li M Lindberg MR Kennett MJ Xiong N Wang Y. PAD4 is Essential for Antibacterial Innate Immunity Mediated by Neutrophil Extracellular Traps. J Exp Med (2010) 207:1853–62. 10.1084/jem.20100239 PMC2931169 20733033

[86] Witalison EE Thompson PR Hofseth LJ. Protein Arginine Deiminases and Associated Cirtullination: Physiological Functions and Diseases Associated With Dysregulation. Curr Drug Targets (2015) 16:700–10. 10.2174/1389450116666150202160954 PMC4520219 25642720

[87] Chang X Han J Pang L Zhao Y Yang Y Shen Z. Increased PADI4 Expression in Blood and Tissues of Patients With Malignant Tumors. BMC Cancer (2009) 9:1–11. 10.1186/1471-2407-9-40 19183436 PMC2637889

[88] Wright PW Bolling LC Calvert ME Sarmento OF Berkeley EV Shea MC. ePAD, An Oocyte and Early Embryo-Abundant Peptidylarginine Deiminase-Like Protein That Localizes to Egg Cytoplasmic Sheets. Dev Biol (2003) 256:74–89. 10.1016/S0012-1606(02)00126-4 12654293

[89] Raijmakers R Zendman AJW Egberts WV Vossenaar ER Raats J Soede-huijbregts C. Methylation of Arginine Residues Interferes With Citrullination by Peptidylarginine Deiminases In Vitro. J Mol Biol (2007) 1:1118–29. 10.1016/j.jmb.2007.01.054 17303166

[90] Esposito G Vitale AM Leijten FPJ Strik AM Koonen-Reemst AMCB Yurttas P. Peptidylarginine Deiminase (PAD) 6 Is Essential for Oocyte Cytoskeletal Sheet Formation and Female Fertility. Mol Cell Endocrinol (2007) 273:25–31. 10.1016/j.mce.2007.05.005 17587491

[91] Tarcsa E Marekov LN Mei G Melino G Lee SC Steinert PM. Protein Unfolding by Peptidylarginine Deiminase: Substrate Specificity and Structural Relationships of the Natural Substrates Trichohyalin and Filaggrin. J Biol Chem (1996) 271:30709–16. 10.1074/jbc.271.48.30709 8940048

[92] Assohou-Luty C Raijmakers R Benckhuijsen WE Stammen-Vogelzangs J De Ru A Van Veelen PA. The Human Peptidylarginine Deiminases Type 2 and Type 4 Have Distinct Substrate Specificities. Biochim Biophys Acta - Proteins Proteomics (2014) 1844:829–36. 10.1016/j.bbapap.2014.02.019 24594197

[93] Hensen SMM Pruijn GJM. Methods for the Detection of Peptidylarginine Deiminase (PAD) Activity and Protein Citrullination. Mol Cell Proteomics (2014) 13:388–96. 10.1074/mcp.R113.033746 PMC3916641 24298040

[94] Damgaard D Senolt L Nielsen MF Pruijn GJ Nielsen CH. Demonstration of Extracellular Peptidylarginine Deiminase (PAD) Activity in Synovial Fluid of Patients With Rheumatoid Arthritis Using a Novel Assay for Citrullination of Fibrinogen. Arthritis Res Ther (2014) 16:498. 10.1186/s13075-014-0498-9 25475141 PMC4298085

[95] Arita K Hashimoto H Shimizu T Nakashima K Yamada M Sato M. Structural Basis for Ca2+-Induced Activation of Human PAD4. Nat Struct Mol Biol (2004) 11:777–83. 10.1038/nsmb799 15247907

[96] Liu GY Liu YL Lee CY Huang YN Chen HY Hung HC. Probing the Roles of Calcium-Binding Sites During the Folding of Human Peptidylarginine Deiminase. Sci Rep (2017) 7:1–14. 10.1038/s41598-017-02677-1 28546558 PMC5445078

[97] Wiik AS van Venrooij WJ Pruijn GJM. All You Wanted to Know About anti-CCP But Were Afraid to Ask. Autoimmun Rev (2010) 10:90–3. 10.1016/j.autrev.2010.08.009 20727426

[98] Machold KP Stamm TA Nell VPK Pflugbeil S Aletaha D Steiner G. Very Recent Onset Rheumatoid Arthritis: Clinical and Serological Patient Characteristics Associated With Radiographic Progression Over the First Years of Disease. Rheumatology (2007) 46:342–9. 10.1093/rheumatology/kel237 16899498

[99] Senshu T Sato T Inoue T Akiyama K Asaga H. Detection of Citrulline Residues in Deiminated Proteins on Polyvinylidene Difluoride Membrane. Anal Biochem (1992) 203:94–100. 10.1016/0003-2697(92)90047-B 1524220

[100] Moelants EAV van Damme J Proost P. Detection and Quantification of Citrullinated Chemokines. PloS One (2011) 6:6–13. 10.1371/journal.pone.0028976 PMC3241686 22194966

[101] Bicker KL Subramanian V Chumanevich AA Hofseth LJ Thompson PR. Seeing Citrulline: Development of a Phenylglyoxal-Based Probe To Visualize Protein Citrullination. J Am Chem Soc (2012) 134:17015–8. 10.1021/ja308871v PMC3572846 23030787

[102] Lewallen DM Bicker KL Subramanian V Clancy KW Slade DJ Martell J. Chemical Proteomic Platform to Identify Citrullinated Proteins. ACS Chem Biol (2015) 10:2520–8. 10.1021/acschembio.5b00438 PMC4729336 26360112

[103] Nicholas AP Sambandam T Echols JD Barnum SR. Expression of Citrullinated Proteins in Murine Experimental Autoimmune Encephalomyelitis. J Comp Neurol (2005) 486:254–66. 10.1002/cne.20527 15844173

[104] Zhou Y Di Pucchio T Sims GP Mittereder N Mustelin T. Characterization of the Hypercitrullination Reaction in Human Neutrophils and Other Leukocytes. Mediators Inflammation (2015) 2015:236451. 10.1155/2015/236451 PMC4452437 26078491

[105] Verheul MK van Veelen PA van Delft MAM de Ru A Janssen GMC Rispens T. Pitfalls in the Detection of Citrullination and Carbamylation. Autoimmun Rev (2018) 17:136–41. 10.1016/j.autrev.2017.11.017 29203292

[106] Clancy KW Weerapana E Thompson PR. Detection and Identification of Protein Citrullination in Complex Biological Systems. Curr Opin Chem Biol (2016) 30:1–6. 10.1016/j.cbpa.2015.10.014 26517730 PMC4731267

[107] Wang X Swensen AC Zhang T Piehowski PD Gaffrey MJ Monroe ME. Accurate Identification of Deamidation and Citrullination From Global Shotgun Proteomics Data Using a Dual-Search Delta Score Strategy. J Proteome Res (2020) 19:1863–72. 10.1021/acs.jproteome.9b00766 PMC7217331 32175737

[108] Holm A Rise F Sessler N Sollid LM Undheim K Fleckenstein B. Specific Modification of Peptide-Bound Citrulline Residues. Anal Biochem (2006) 352:68–76. 10.1016/j.ab.2006.02.007 16540076

[109] De Ceuleneer M De Wit V Van Steendam K Van Nieuwerburgh F Tilleman K Deforce D. Modification of Citrulline Residues With 2,3-Butanedione Facilitates Their Detection by Liquid Chromatography/Mass Spectrometry. Rapid Commun Mass Spectrom (2011) 25:1536–42. 10.1002/rcm.5015 21594927

[110] Tilvawala R Nguyen SH Maurais AJ Nemmara VV Nagar M Salinger AJ. The Rheumatoid Arthritis-Associated Citrullinome. Cell Chem Biol (2018) 25:691–704.e6. 10.1016/j.chembiol.2018.03.002 29628436 PMC6014894

[111] Zullo A Sommese L Nicoletti G Donatelli F Mancini FP Napoli C. Epigenetics and Type 1 Diabetes: Mechanisms and Translational Applications. Transl Res (2017) 185:85–93. 10.1016/j.trsl.2017.05.002 28552218

[112] Rohrbach AS Slade DJ Thompson PR Mowen KA. Activation of PAD4 in NET Formation. Front Immunol (2012) 3:360. 10.3389/fimmu.2012.00360 23264775 PMC3525017

[113] de Bont CM Koopman WJH Boelens WC Pruijn GJM. Stimulus-Dependent Chromatin Dynamics, Citrullination, Calcium Signalling and ROS Production During NET Formation. Biochim Biophys Acta - Mol Cell Res (2018) 1865:1621–9. 10.1016/j.bbamcr.2018.08.014 30327203

[114] Damgaard D Bjørn ME Jensen PØ Nielsen CH. Reactive Oxygen Species Inhibit Catalytic Activity of Peptidylarginine Deiminase. J Enzyme Inhib Med Chem (2017) 32:1203–8. 10.1080/14756366.2017.1368505 PMC6021033 28933232

[115] Nagar M Tilvawala R Thompson PR. Thioredoxin Modulates Protein Arginine Deiminase 4. Front Immunol (2019) 10:244. 10.3389/fimmu.2019.00244 30853960 PMC6396667

[116] Damgaard D Bjørn ME Steffensen MA Pruijn GJM Nielsen CH. Reduced Glutathione as a Physiological Co-Activator in the Activation of Peptidylarginine Deiminase. Arthritis Res Ther (2016) 18:1–7. 10.1186/s13075-016-1000-7 27149996 PMC4858833

[117] Eizirik DL Colli ML Ortis F. The Role of Inflammation in Insulitis and β-Cell Loss in Type 1 Diabetes. Nat Rev Endocrinol (2009) 5:219–26. 10.1038/nrendo.2009.21 19352320

[118] Vig S Buitinga M Rondas D Crèvecoeur I van Zandvoort M Waelkens E. Cytokine-Induced Translocation of GRP78 to the Plasma Membrane Triggers a Pro-Apoptotic Feedback Loop in Pancreatic Beta Cells. Cell Death Dis (2019) 10:309. 10.1038/s41419-019-1518-0 30952835 PMC6450900

[119] Eizirik DL Sammeth M Bouckenooghe T Bottu G Sisino G Igoillo-Esteve M. The Human Pancreatic Islet Transcriptome: Expression of Candidate Genes for Type 1 Diabetes and the Impact of Pro-Inflammatory Cytokines. PloS Gene (2012) 8:e1002552. 10.1371/journal.pgen.1002552 PMC3297576 22412385

[120] Schuit FC In ‘t Veld PA Pipeleers DG. Glucose Stimulates Proinsulin Biosynthesis by a Dose-Dependent Recruitment of Pancreatic Beta Cells. Proc Natl Acad Sci USA (1988) 85:3865–9. 10.1073/pnas.85.11.3865 PMC280320 3287379

[121] Scheuner D Kaufman RJ. The Unfolded Protein Response: A Pathway That Links Insulin Demand With β-Cell Failure and Diabetes. Endocr Rev (2008) 29:317–33. 10.1210/er.2007-0039 PMC2528859 18436705

[122] Hetz C Papa FR. The Unfolded Protein Response and Cell Fate Control. Mol Cell (2018) 69:169–81. 10.1016/j.molcel.2017.06.017 29107536

[123] Grootjans J Kaser A Kaufman RJ Blumberg RS. The Unfolded Protein Response in Immunity and Inflammation. Nat Rev Immunol (2016) 16:469–84. 10.1038/nri.2016.62 PMC5310224 27346803

[124] Ghosh R Colon-Negron K Papa FR. Endoplasmic Reticulum Stress, Degeneration of Pancreatic Islet β-Cells, and Therapeutic Modulation of the Unfolded Protein Response in Diabetes. Mol Metab (2019) 27:S60–8. 10.1016/j.molmet.2019.06.012 PMC6768499 31500832

[125] Marré ML James EA Piganelli JD. β Cell ER Stress and the Implications for Immunogenicity in Type 1 Diabetes. Front Cell Dev Biol (2015) 3:67. 10.3389/fcell.2015.00067 26579520 PMC4621612

[126] Marré ML Piganelli JD. Environmental Factors Contribute to β Cell Endoplasmic Reticulum Stress and Neo-Antigen Formation in Type 1 Diabetes. Front Endocrinol (Lausanne) (2017) 8:262. 10.3389/fendo.2017.00262 29033899 PMC5626851

[127] Marre ML Mcginty J Chow I-T Denicola ME Beck NW Kent SC. Modifying Enzymes are Elicited by ER Stress, Generating Epitopes That Are Selectively Recognized by CD4 + T Cells in Patients With Type 1 Diabetes. Diabetes (2018) 67:1356–68. 10.2337/db17-1166 PMC6014552 29654212

[128] Marré ML Profozich JL Coneybeer JT Geng X Bertera S Ford MJ. Inherent ER Stress in Pancreatic Islet β Cells Causes Self-Recognition by Autoreactive T Cells in Type 1 Diabetes. J Autoimmun (2016) 72:33–46. 10.1016/j.jaut.2016.04.009 27173406 PMC4958612

[129] D’Hertog W Overbergh L Lage K Ferreira GB Maris M Gysemans C. Proteomics Analysis of Cytokine-Induced Dysfunction and Death in Insulin-Producing INS-1E Cells: New Insights Into the Pathways Involved. Mol Cell Proteomics (2007) 6:2180–99. 10.1074/mcp.M700085-MCP200 17921177

[130] Christophorou MA Castelo-Branco G Halley-Stott RP Oliveira CS Loos R Radzisheuskaya A. Citrullination Regulates Pluripotency and Histone H1 Binding to Chromatin. Nature (2014) 507:104–8. 10.1038/nature12942 PMC4843970 24463520

[131] Slade DJ Subramanian V Thompson PR. Pluripotency: Citrullination Unravels Stem Cells. Nat Chem Biol (2014) 10:327–8. 10.1038/nchembio.1504 PMC4632640 24743255

[132] Fuhrmann J Thompson PR. Protein Arginine Methylation and Citrullination in Epigenetic Regulation. ACS Chem Biol (2016) 11:654–68. 10.1021/acschembio.5b00942 PMC4802296 26686581

[133] Zhang X Liu X Zhang M Li T Muth A Thompson PR. Peptidylarginine Deiminase 1-Catalyzed Histone Citrullination Is Essential for Early Embryo Development. Sci Rep (2016) 6:1–11. 10.1038/srep38727 27929094 PMC5144008

[134] Wang Y Li M Stadler S Correll S Li P Wang D. Histone Hypercitrullination Mediates Chromatin Decondensation and Neutrophil Extracellular Trap Formation. J Cell Biol (2009) 184:205–13. 10.1083/jcb.200806072 PMC2654299 19153223

[135] Konig MF Andrade F. A Critical Reappraisal of Neutrophil Extracellular Traps and NETosis Mimics Based on Differential Requirements for Protein Citrullination. Front Immunol (2016) 7:461. 10.3389/fimmu.2016.00461 27867381 PMC5095114

[136] Sharma P Azebi S England P Christensen T Møller-Larsen A Petersen T. Citrullination of Histone H3 Interferes With HP1-Mediated Transcriptional Repression. PloS Genet (2012) 8:1–15. 10.1371/journal.pgen.1002934 PMC3441713 23028349

[137] Guertin MJ Zhang X Anguish L Kim S Varticovski L Lis JT. Targeted H3R26 Deimination Specifically Facilitates Estrogen Receptor Binding by Modifying Nucleosome Structure. PloS Genet (2014) 10:1–12. 10.1371/journal.pgen.1004613 PMC4161307 25211228

[138] Perri AM Agosti V Olivo E Concolino A Angelis M Tammè L. Histone Proteomics Reveals Novel Post-Translational Modifications in Breast Cancer. Aging (Albany NY) (2019) 11:11722–55. 10.18632/aging.102577 PMC6932915 31816600

[139] Tee WW Pardo M Theunissen TW Yu L Choudhary JS Hajkova P. Prmt5 is Essential for Early Mouse Development and Acts in the Cytoplasm to Maintain ES Cell Pluripotency. Genes Dev (2010) 24:2772–7. 10.1101/gad.606110 PMC3003195 21159818

[140] Deng X Shao G Zhang HT Li C Zhang D Cheng L. Protein Arginine Methyltransferase 5 Functions as an Epigenetic Activator of the Androgen Receptor to Promote Prostate Cancer Cell Growth. Oncogene (2017) 36:1223–31. 10.1038/onc.2016.287 PMC5322258 27546619

[141] Cherrington BD Zhang X McElwee JL Morency E Anguish LJ Coonrod SA. Potential Role for PAD2 in Gene Regulation in Breast Cancer Cells. PloS One (2012) 7:1–12. 10.1371/journal.pone.0041242 PMC3404060 22911765

[142] Delgado-Rizo V Martínez-Guzmán MA Iñiguez-Gutierrez L García-Orozco A Alvarado-Navarro A Fafutis-Morris M. Neutrophil Extracellular Traps and Its Implications in Inflammation: An Overview. Front Immunol (2017) 8:81. 10.3389/fimmu.2017.00081 28220120 PMC5292617

[143] Lewis HD Liddle J Coote JE Atkinson SJ Barker MD Bax BD. Inhibition of PAD4 Activity is Sufficient to Disrupt Mouse and Human NET Formation. Nat Chem Biol (2015) 11:181–91. 10.1038/nchembio.1735 PMC4397581 25622091

[144] Khandpur R Carmona-rivera C Vivekanandan-giri A Gizinski A Yalavarthi S Knight JS. Nets Are a Source of Citrullinated Autoantigens and Stimulate Inflammatory Responses in Rheumatoid Arthritis. Rheum Arthritis (2013) 5:1–10. 10.1126/scitranslmed.3005580 PMC3727661 23536012

[145] Pratesi F Dioni I Tommasi C Alcaro MC Paolini I Barbetti F. Antibodies From Patients With Rheumatoid Arthritis Target Citrullinated Histone 4 Contained in Neutrophils Extracellular Traps. Ann Rheum Dis (2014) 73:1414–22. 10.1136/annrheumdis-2012-202765 23727635

[146] Diana J Simoni Y Furio L Beaudoin L Agerberth B Barrat F. Crosstalk Between Neutrophils, B-1a Cells and Plasmacytoid Dendritic Cells Initiates Autoimmune Diabetes. Nat Med (2013) 19:65–73. 10.1038/nm.3042 23242473

[147] Qin J Fu S Speake C Greenbaum CJ Odegard JM. Netosis-Associated Serum Biomarkers Are Reduced in Type 1 Diabetes in Association With Neutrophil Count. Clin Exp Immunol (2016) 184:318–22. 10.1111/cei.12783 PMC4872375 26939803

[148] Wang Y Xiao Y Zhong L Ye D Zhang J Tu Y. Increased Neutrophil Elastase and Proteinase 3 and Augmented NETosis Are Closely Associated With β-Cell Autoimmunity in Patients With Type 1 Diabetes. Diabetes (2014) 63:4239–48. 10.2337/db14-0480 25092677

[149] Harsunen MH Puff R D’Orlando O Giannopoulou E Lachmann L Beyerlein A. Reduced Blood Leukocyte and Neutrophil Numbers in the Pathogenesis of Type 1 Diabetes. Horm Metab Res (2013) 45:467–70. 10.1055/s-0032-1331226 23322517

[150] Valle A Giamporcaro GM Scavini M Stabilini A Grogan P Bianconi E. Reduction of Circulating Neutrophils Precedes and Accompanies Type 1 Diabetes. Diabetes (2013) 62:2072–7. 10.2337/db12-1345 PMC3661622 23349491

[151] Vecchio F Buono N Stabilini A Nigi L Dufort MJ Geyer S. Abnormal Neutrophil Signature in the Blood and Pancreas of Presymptomatic and Symptomatic Type 1 Diabetes. JCI Insight (2018) 3:1–17. 10.1172/JCI.INSIGHT.122146 PMC6237216 30232284

[152] Ling Wong S Demers M Martinod K Gallant M Wang Y Goldfine ABB. Diabetes Primes Neutrophils to Undergo NETosis, Which Impairs Wound Healing. Nat Med (2015) 21:815–9. 10.1038/nm.3887 PMC4631120 26076037

[153] El Shikh MEM El Sayed R Nerviani A Goldmann K John CR Hands R. Extracellular Traps and PAD4 Released by Macrophages Induce Citrullination and Auto-Antibody Production in Autoimmune Arthritis. J Autoimmun (2019) 105:102297. 10.1016/j.jaut.2019.06.008 31277965

[154] Sharma P Lioutas A Fernandez-Fuentes N Quilez J Carbonell-Caballero J Wright RHG. Arginine Citrullination At the C-Terminal Domain Controls Rna Polymerase II Transcription. Mol Cell (2019) 73:84–96.e7. 10.1016/j.molcel.2018.10.016 30472187

[155] Proost P Loos T Mortier A Schutyser E Gouwy M Noppen S. Citrullination of CXCL8 by Peptidylarginine Deiminase Alters Receptor Usage, Prevents Proteolysis, and Dampens Tissue Inflammation. J Exp Med (2008) 205:2085–97. 10.1084/jem.20080305 PMC2526203 18710930

[156] Loos T Mortier A Gouwy M Ronsse I Put W Lenaerts JP. Citrullination of CXCL10 and CXCL11 by Peptidylarginine Deiminase: A Naturally Occurring Posttranslational Modification of Chemokines and New Dimension of Immunoregulation. Blood (2008) 112:2648–56. 10.1182/blood-2008-04-149039 18645041

[157] Struyf S Noppen S Loos T Mortier A Gouwy M Verbeke H. Citrullination of CXCL12 Differentially Reduces CXCR4 and CXCR7 Binding With Loss of Inflammatory and Anti-HIV-1 Activity Via CXCR4. J Immunol (2009) 182:666–74. 10.4049/jimmunol.182.1.666 19109200

[158] Moelants EAV Mortier A Grauwen K Ronsse I Van Damme J Proost P. Citrullination of TNF-α by Peptidylarginine Deiminases Reduces its Capacity to Stimulate the Production of Inflammatory Chemokines. Cytokine (2013) 61:161–7. 10.1016/j.cyto.2012.09.011 23075670

[159] Kondo K Ohigashi I Takahama Y. Thymus Machinery for T-cell Selection. Int Immunol (2019) 31:119–25. 10.1093/intimm/dxy081 PMC6400048 30476234

[160] Derbinski J Schulte A Kyewski B Klein L. Promiscuous Gene Expression in Medullary Thymic Epithelial Cells Mirrors the Peripheral Self. J Immunol (2001) 2:1032–9. 10.1038/ni723 11600886

[161] Delong T Baker RL Reisdorph N Reisdorph R Powell RL Armstrong M. Islet Amyloid Polypeptide is a Target Antigen for Diabetogenic CD4 + T Cells. Diabetes (2011) 60:2325–30. 10.2337/db11-0288 PMC3161333 21734016

[162] Raposo B Merky P Lundqvist C Yamada H Urbonaviciute V Niaudet C. T Cells Specific for Post-Translational Modifications Escape Intrathymic Tolerance Induction. Nat Commun (2018) 9:353. 10.1038/s41467-017-02763-y 29367624 PMC5783942

[163] Engelmann R Biemelt A Cordshagen A Johl A Kuthning D Müller-Hilke B. The Prerequisites for Central Tolerance Induction Against Citrullinated Proteins in the Mouse. PloS One (2016) 11:1–11. 10.1371/journal.pone.0158773 PMC4928850 27362943

[164] Rodriguez-Calvo T Johnson JD Overbergh L Dunne JL. Neoepitopes in Type 1 Diabetes: Etiological Insights, Biomarkers and Therapeutic Targets. Front Immunol (2021) 12:667989. 10.3389/fimmu.2021.667989 33953728 PMC8089389

[165] Crèvecoeur I Gudmundsdottir V Vig S Marques Câmara Sodré F D’Hertog W Fierro AC. Early Differences in Islets From Prediabetic NOD Mice: Combined Microarray and Proteomic Analysis. Diabetologia (2017) 60:475–89. 10.1007/s00125-016-4191-1 28078386

[166] Hayashi H Morioka M Ichimiya S Yamato K Hinode D Nagata A. Nakamura R. Participation of an Arginyl Residue of Insulin Chain B in the Inhibition of Hemagglutination by Porphyromonas Gingivalis. Oral Microbiol Immunol (1993) 8:386–9. 10.1111/j.1399-302X.1993.tb00616.x 8152841

[167] Nguyen H James EA. Immune Recognition of Citrullinated Epitopes. Immunology (2016) 149:131–8. 10.1111/imm.12640 PMC5011684 27531825

[168] Mamula MJ Gee RJ Elliott JI Sette A Southwood S Jones PJ. Isoaspartyl Post-Translational Modification Triggers Autoimmune Responses to Self-Proteins. J Biol Chem (1999) 274:22321–7. 10.1074/jbc.274.32.22321 10428801

[169] Yang M-LL Doyle HA Clarke SG Herold KC Mamula MJ. Oxidative Modifications in Tissue Pathology and Autoimmune Disease. Antioxid Redox Signal (2018) 29:1415–31. 10.1089/ars.2017.7382 PMC6166690 29088923

[170] Lehmann PV Forsthuber T Miller A Sercarz EE. Spreading of T-cell Autoimmunity to Cryptic Determinants of an Autoantigen. Nature (1992) 358:155–7. 10.1038/358155a0 1377368

[171] Doyle HA Aswad DW Mamula MJ. Autoimmunity to Isomerized Histone H2B in Systemic Lupus Erythematosus. Autoimmunity (2013) 46:6–13. 10.3109/08916934.2012.710859 22967069 PMC3543506

[172] Kidd BA Ho PP Sharpe O Zhao X Tomooka BH Kanter JL. Epitope Spreading to Citrullinated Antigens in Mouse Models of Autoimmune Arthritis and Demyelination. Arthritis Res Ther (2008) 10:1–12. 10.1186/ar2523 PMC2592807 18826638

[173] Arbuckle MR McClain MT Rubertone MV Scofield RH Dennis GJ James JA. Development of Autoantibodies Before the Clinical Onset of Systemic Lupus Erythematosus. N Engl J Med (2003) 349:1526–33. 10.1056/nejmoa021933 14561795

[174] Gergely P Grossman C Niland B Puskas F Neupane H Allam F. Mitochondrial Hyperpolarization and ATP Depletion in Patients With Systemic Lupus Erythematosus. Arthritis Rheum (2002) 46:175–90. 10.1002/1529-0131(200201)46:1<175::AID-ART10015>3.0.CO;2-H PMC4020417 11817589

[175] Gergely P Niland B Gonchoroff N Pullmann R Phillips PE Perl A. Persistent Mitochondrial Hyperpolarization, Increased Reactive Oxygen Intermediate Production, and Cytoplasmic Alkalinization Characterize Altered IL-10 Signaling in Patients With Systemic Lupus Erythematosus. J Immunol (2002) 169:1092–101. 10.4049/jimmunol.169.2.1092 PMC4020441 12097418

[176] Becart S Whittington KB Prislovsky A Rao NL Rosloniec F E. The Role of Posttranslational Modifications in Generating Neo-Epitopes That Bind to Rheumatoid Arthritis-Associated HLA-DR Alleles and Promote Autoimmune T Cell Responses. PloS One (2021) 16:1–22. 10.1371/journal.pone.0245541 PMC7815092 33465118

[177] Travers TS Harlow L Rosas IO Gochuico BR Mikuls TR Bhattacharya SK. Extensive Citrullination Promotes Immunogenicity of HSP90 Through Protein Unfolding and Exposure of Cryptic Epitopes. J Immunol (2016) 197:1926–36. 10.4049/jimmunol.1600162 PMC5061338 27448590

[178] Manoury B Mazzeo D Fugger L Viner N Ponsford M Streeter H. Destructive Processing by Asparagine Endopeptidase Limits Presentation of a Dominant T Cell Epitope in MBP. Nat Immunol (2002) 3:169–74. 10.1038/ni754 11812994

[179] Gahring LC Carlson NG Meyer EL Rogers SW. Cutting Edge: Granzyme B Proteolysis of a Neuronal Glutamate Receptor Generates an Autoantigen and Is Modulated by Glycosylation. J Immunol (2001) 166:1433–8. 10.4049/jimmunol.166.3.1433 11160179

[180] Johnson BA Aswad DW. Fragmentation of Isoaspartyl Peptides and Proteins by Carboxypeptidase Y: Release of Isoaspartyl Dipeptides as a Result of Internal and External Cleavage. Biochemistry (1990) 29:4373–80. 10.1021/bi00470a017 2140948

[181] Moss CX Matthews SP Lamont DJ Watts C. Asparagine Deamidation Perturbs Antigen Presentation on Class II Major Histocompatibility Complex Molecules. J Biol Chem (2005) 280:18498–503. 10.1074/jbc.M501241200 15749706

[182] Mamula MJ. The Inability to Process a Self-Peptide Allows Autoreactive T Cells to Escape Tolerance. J Exp Med (1993) 177:567–71. 10.1084/jem.177.2.567 PMC2190887 8381158

[183] Mamula MJ. Lupus Autoimmunity; From Peptides to Particles. Immunol Rev (1995) 144:301–14. 10.1111/j.1600-065X.1995.tb00074.x 7590818

[184] Mamula MJ. Epitope Spreading: The Role of Self Peptides and Autoantigen Processing by B Lymphocytes. Immunol Rev (1998) 164:231–3. 10.1111/j.1600-065X.1998.tb01223.x 9795779

[185] Casciola-Rosen LA Miller DK Anhalt GJ Rosen A. Specific Cleavage of the 70-kDa Protein Component of the U1 Small Nuclear Ribonucleoprotein is a Characteristic Biochemical Feature of Apoptotic Cell Death. J Biol Chem (1994) 269:30757–60. 10.1016/s0021-9258(18)47343-7 7983001

[186] Ireland JM Unanue ER. Autophagy in Antigen-Presenting Cells Results in Presentation of Citrullinated Peptides to CD4 T Cells. J Exp Med (2011) 208:2625–32. 10.1084/jem.20110640 PMC3244027 22162830

[187] Harvey BP Gee RJ Haberman AM Shlomchik MJ Mamula MJ. Antigen Presentation and Transfer Between B Cells and Macrophages. Eur J Immunol (2007) 37:1739–51. 10.1002/eji.200636452 17534863

[188] Harvey BP Quan TE Rudenga BJ Roman RM Craft J Mamula MJ. Editing Antigen Presentation: Antigen Transfer Between Human B Lymphocytes and Macrophages Mediated by Class A Scavenger Receptors. J Immunol (2008) 181:4043–51. 10.4049/jimmunol.181.6.4043 PMC2701691 18768860

[189] Raycroft MT Harvey BP Bruck MJ Mamula MJ. Inhibition of Antigen Trafficking Through Scavenger Receptor A. J Biol Chem (2012) 287:5310–6. 10.1074/jbc.M111.316356 PMC3285311 22215667

[190] Hill JA Southwood S Sette A Jevnikar AM Bell DA Cairns E. The Conversion of Arginine to Citrulline Allows for a High-Affinity Peptide Interaction With the Rheumatoid Arthritis-Associated HLA-DRB1*0401 Mhc Class II Molecule. J Immunol (2003) 171:538–41. 10.4049/jimmunol.171.2.538 12847215

[191] Haan ECD Wagenaar-Hilbers JPA Liskamp RMJ Moret EE Wauben MHM. Limited Plasticity in T Cell Recognition of Modified T Cell Receptor Contact Residues in MHC Class II Bound Peptides. Mol Immunol (2005) 42:355–64. 10.1016/j.molimm.2004.07.044 15589324

[192] Slade DJ Fang P Dreyton CJ Zhang Y Fuhrmann J Rempel D. Protein Arginine Deiminase 2 Binds Calcium in an Ordered Fashion: Implications for Inhibitor Design. ACS Chem Biol (2015) 10:1043–53. 10.1021/cb500933j PMC4569063 25621824

[193] Stone EM Schaller TH Bianchi H Person MD Fast W. Inactivation of Two Diverse Enzymes in the Amidinotransferase Superfamily by 2-Chloroacetamidine: Dimethylargininase and Peptidylarginine Deiminase. Biochemistry (2005) 44:13744–52. 10.1021/bi051341y 16229464

[194] Muth A Subramanian V Beaumont E Nagar M Kerry P Mcewan P. Development of a Selective Inhibitor of Protein Arginine Deiminase 2. J Med Chem (2017) 60:3198–211. 10.1021/acs.jmedchem.7b00274 PMC5477668 28328217

[195] Mondal S Thompson PR. Protein Arginine Deiminases (Pads): Biochemistry and Chemical Biology of Protein Citrullination. Acc Chem Res (2019) 52:818–32. 10.1021/acs.accounts.9b00024 PMC6443095 30844238

[196] Johnson DS Weerapana E Cravatt BF. Strategies for Discovering and Derisking Covalent, Irreversible Enzyme Inhibitors. Future Med Chem (2010) 2:949–64. 10.4155/fmc.10.21 PMC2904065 20640225

[197] Nemmara VV Thompson PR. Development of Activity-Based Proteomic Probes for Protein Citrullination. Curr Top Microbiol Immunol (2019) 420:233–51. 10.1007/82 PMC6348022 30203394

[198] Lewis HD Nacht M. IPAD or PADi - “Tablets” With Therapeutic Disease Potential? Curr Opin Chem Biol (2016) 33:169–78. 10.1016/j.cbpa.2016.06.020 27372273

[199] Bruggeman Y Sodré FMC Buitinga M Mathieu C Kracht MJL. Expert Opinion on Therapeutic Targets Targeting Citrullination in Autoimmunity: Insights Learned From Preclinical Mouse Models Models. Expert Opin Ther Targets (2021), 1–13. 10.1080/14728222.2021.1918104 33896351

[200] Sodré FMC Bissenova S Bruggeman Y Tilvawala R Cook DP Berthault C. Peptidylarginine Deiminase Inhibition Prevents Diabetes Development in NOD Mice. Diabetes (2021) 70:516–28. 10.2337/db20-0421 PMC7881854 33203696

[201] Kawalkowska J Quirke AM Ghari F Davis S Subramanian V Thompson PR. Abrogation of Collagen-Induced Arthritis by a Peptidyl Arginine Deiminase Inhibitor Is Associated With Modulation of T Cell-Mediated Immune Responses. Sci Rep (2016) 6:1–12. 10.1038/srep26430 27210478 PMC4876390

[202] Chumanevich AA Causey CP Knuckley BA Jones JE Poudyal D Chumanevich AP. Suppression of Colitis in Mice by Cl-Amidine: A Novel Peptidylarginine Deiminase Inhibitor. Am J Physiol - Gastrointest Liver Physiol (2011) 300:929–38. 10.1152/ajpgi.00435.2010 PMC3119113 21415415

[203] Willis VC Gizinski AM Banda NK Causey CP Knuckley B Cordova KN. N-α-Benzoyl-N5-(2-Chloro-1-Iminoethyl)-L-Ornithine Amide, A Protein Arginine Deiminase Inhibitor, Reduces the Severity of Murine Collagen-Induced Arthritis. J Immunol (2011) 186:4396–404. 10.4049/jimmunol.1001620 PMC3085980 21346230

[204] Knight JS Zhao W Luo W Subramanian V Dell AAO Yalavarthi S. Peptidylarginine Deiminase Inhibition Is Immunomodulatory and Vasculoprotective in Murine Lupus. J Clin Invest (2013) 123:2981–93. 10.1172/JCI67390.ex PMC3696545 23722903

[205] Knight JS Subramanian V O’Dell AA Yalavarthi S Zhao W Smith CK. Peptidylarginine Deiminase Inhibition Disrupts NET Formation and Protects Against Kidney, Skin and Vascular Disease in Lupus-Prone MRL/ Lpr Mice. Ann Rheum Dis (2015) 74:2199–206. 10.1136/annrheumdis-2014-205365 PMC4320672 25104775

[206] Kawaguchi H Matsumoto I Osada A Kurata I Ebe H Tanaka Y. Peptidyl Arginine Deiminase Inhibition Suppresses Arthritis Via Decreased Protein Citrullination in Joints and Serum With the Downregulation of Interleukin-6. Mod Rheumatol (2019) 29:964–9. 10.1080/14397595.2018.1532545 30285515

[207] Ghari F Quirke AM Munro S Kawalkowska J Picaud S McGouran J. Citrullination-Acetylation Interplay Guides E2F-1 Activity During the Inflammatory Response. Sci Adv (2016) 2:e1501257. 10.1126/sciadv.1501257 26989780 PMC4788482

[208] Papadaki G Kambas K Choulaki C Vlachou K Drakos E Bertsias G. Neutrophil Extracellular Traps Exacerbate Th1-Mediated Autoimmune Responses in Rheumatoid Arthritis by Promoting DC Maturation. Eur J Immunol (2016) 46:2542–54. 10.1002/eji.201646542 PMC5476297 27585946

[209] Luo Y Arita K Bhatia M Knuckley B Lee YH Stallcup MR. Inhibitors and Inactivators of Protein Arginine Deiminase 4: Functional and Structural Characterization. Biochemistry (2006) 45:11727–36. 10.1021/bi061180d PMC1808342 17002273

[210] Makrygiannakis D Af Klint E Lundberg IE Löfberg R Ulfgren AK Klareskog L. Citrullination Is an Inflammation-Dependent Process. Ann Rheum Dis (2006) 65:1219–22. 10.1136/ard.2005.049403 PMC1798285 16540548

[211] Bicker KL Anguish L Chumanevich AA Cameron MD Cui X Witalison E. D-Amino Acid-Based Protein Arginine Deiminase Inhibitors: Synthesis, Pharmacokinetics, and in Cellulo Efficacy. ACS Med Chem Lett (2012) 3:1081–5. 10.1021/ml300288d PMC3572853 23420624

[212] Jones JE Slack JL Fang P Zhang X Subramanian V Causey CP. Synthesis and Screening of a Haloacetamidine Containing Library to Identify PAD4 Selective Inhibitors. ACS Chem Biol (2012) 7:160–5. 10.1021/cb200258q PMC3262960 22004374

[213] Willis VC Banda NK Cordova KN Chandra PE Robinson WH Cooper DC. Protein Arginine Deiminase 4 Inhibition Is Sufficient for the Amelioration of Collagen-Induced Arthritis. Clin Exp Immunol (2017) 188:263–74. 10.1111/cei.12932 PMC5383440 28128853

